# Hypoxia-Related Signature Is a Prognostic Biomarker of Pancreatic Cancer

**DOI:** 10.1155/2022/6449997

**Published:** 2022-06-25

**Authors:** Jing-jing Zhang, Chao Shao, Yi-xin Yin, Qiang Sun, Ya-ni Li, Ya-wen Zha, Min-ying Li, Bang-li Hu

**Affiliations:** ^1^Cancer Institute of Zhongshan City People's Hospital, Zhongshan, 528403 Guangdong, China; ^2^Department of Research, Guangxi Medical University Cancer Hospital, Nanning, 530021 Guangxi, China; ^3^Department of Hepatobiliary Surgery, Zhongshan City People's Hospital, Zhongshan, 528403 Guangdong, China

## Abstract

**Background:**

Hypoxia plays a significant role in the pathogenesis of pancreatic cancer, but the effect of hypoxia-related genes in pancreatic cancer remains to be elucidated. This study aimed to identify hypoxia-related genes related to pancreatic cancer and construct a prognostic signature.

**Methods:**

Pancreatic cancer datasets were retrieved from TCGA database. Cox regression analyses were used to identify hypoxia-related genes and construct a prognostic signature. Datasets from International Cancer Genome Consortium and GEO databases were used as validated cohorts. The CIBERSORT method was applied to estimate the fractions of immune cell types. DNA methylation and protein levels of the genes in pancreatic cancer were examined.

**Results:**

Three hypoxia-related genes (TES, LDHA, and ANXA2) were identified as associated with patient survival and selected to construct a prognostic signature. Patients were divided into high- and low-risk groups based on the signature. Those in the high-risk group showed worse survival than those in the low-risk group. The signature was shown to be involved in the HIF-1 signaling pathway. The time-dependent ROC analyses of three independent validated cohorts further revealed that this signature had a better prognostic value in the prediction of the survival of pancreatic cancer patients. Immune cells analysis for three datasets demonstrated that high-risk signature was significantly associated with macrophages and T cells. DNA methylation and protein levels of the three genes validated their aberrant expression in pancreatic cancer.

**Conclusions:**

Our research provided a novel and reliable prognostic signature that composes of three hypoxia-related genes to estimate the prognosis of pancreatic cancer.

## 1. Introduction

Pancreatic cancer is a common, malignant solid carcinoma that poses a great threat to human health [1]. Despite significant advancements have been made in the treatment modalities for pancreatic cancer, the survival of patients with pancreatic cancer remains poor, especially those at the late stage of disease [2]. To date, several molecular markers have been identified for the early diagnosis, treatment monitoring, and survival prediction in pancreatic cancer patients. These have remarkably improved the prognosis of patients and provided new targets for treatment [3]. However, although the molecular mechanism of pancreatic cancer pathogenesis has been studied in past decades, the exact mechanism remains largely unknown and requires further study.

Hypoxia is a key characteristic feature of the tumor microenvironment (TME), mostly occurring in solid tumors [4]. The occurrence of hypoxia in the TME is due to the imbalance between rapid proliferation of tumor cells and insufficient blood and nutrition supply. Under hypoxic conditions, tumors initiate a wide array of adaptive behaviors, including tumor cell epithelial-mesenchymal transition, proliferation, differentiation, and invasion in response to low oxygen levels, and finally leading to a more aggressive nature of the tumor [5]. Hypoxia also occurs in pancreatic cancer, and some hypoxia-related molecules such as ADAM8 [6], BX111 [7], and CF129 [8] have been identified to play crucial roles in the progression of pancreatic cancer, which might have potential as prognostic biomarkers and therapeutic targets for pancreatic cancer. In addition, the complex interplay between immune cell infiltrates within the hypoxic TME of cancers has been reported in several studies. For example, hypoxia was shown to be a dominant remodeler of the effector T cell surface proteome, relative to activation and regulatory T cell suppression [9]. Hypoxia was also found to drive immunosuppression by Treg and type-2 conventional dendritic cells in hepatocellular carcinoma [10]. These results indicated that hypoxia played important roles in the regulation of immune cells in cancers. Thus, exploring the role of hypoxia-related genes or signature genes in cancers and the interaction with immune cells might help to uncover the mechanism of pathogenesis and progression in cancer and predict the survival of patients with cancers.

Some previous studies have constructed prognostic signatures using hypoxia-related genes to predict the prognosis of cancer, including lung cancer [11], glioma [12], and hepatocellular carcinoma [13]. However, prognostic signatures using hypoxia-related genes in patients with pancreatic cancer are still limited [14]. Therefore, in this study, we aimed to identify hypoxia-related genes in pancreatic cancer, constructing a prognostic signature to predict the prognosis of patients with pancreatic cancer. Next, we explored the association between the hypoxia-related prognostic signature and immune infiltration in pancreatic cancer. Finally, we examined the effect of DNA methylation on the expression of hypoxia-related genes and validated the protein expression of genes in pancreatic tissues.

## 2. Materials and Methods

### 2.1. Data Collection

The gene profile dataset of pancreatic cancer (PAAD, level 3 RNA-seq FPKM) was downloaded from The Cancer Genome Atlas (TCGA) database, which included 179 cancer tissues and four normal tissues. Because the number of normal tissues was small, to improve the statistical robustness, we also downloaded a dataset of 167 normal pancreatic tissues from the GTEx database, which was used to combine the data with the normal data of the TCGA-PAAD dataset. The dataset of 189 patients with pancreatic adenocarcinoma was downloaded from the International Cancer Genome Consortium and used as one of the validated datasets (ICGC-PACA). In addition, the GSE57495 dataset [15] (GPL15048 platform, Rosetta/Merck Human RSTA Custom Affymetrix 2.0 microarray) retrieved from the Gene Expression Omnibus (GEO) database was used as another validated dataset, and it included the data of 63 pancreatic cancers. The related clinical data of the three datasets were extracted. The hallmark gene sets of hypoxia were retrieved from Molecular Signatures Database (MSigDB version 6.0), including 200 genes that were found to be upregulated during hypoxia (Table S1).

### 2.2. Identification of Differentially Expressed Genes and Hypoxia-Related Genes for Prognosis

Differentially expressed genes (DEGs) between pancreatic cancer and normal tissues were screened in the TCGA-PAAD dataset using the “edgeR” package and used for examining differential expression of replicated count data [16]. The DEGs were defined as absolute log2-fold change (|FC|) > 1.0 and an adjusted *P* value of less than 0.05. Then, the DEGs between pancreatic cancer and normal tissues were overlapped with the known 200 hypoxia genes from MSigDB; the common genes of the two gene sets were defined as hypoxia-related DEGs. Next, univariate Cox regression analyses were conducted to screen the identify the hypoxia-related DEGs that related to overall survival (OS) of pancreatic cancer patient, and genes with a *P* value < 0.05 were considered statistically significant; finally, the hypoxia-related DEGs from univariate Cox regression analyses were incorporated into multivariate Cox regression analyses for further analysis; genes with a *P* value less than 0.05 were used in the subsequent analysis.

### 2.3. Establishment and Validation of a Prognostic Gene Signature

The hypoxia-related prognostic gene signature was constructed using the hypoxia-related gene from multivariate Cox regression analyses as previously reported [11]. Briefly, the regression coefficient (*β*) value of genes derived from multivariate Cox regression analyses was multiplied by the expression of the corresponding gene to generate the risk score, and the risk score is then calculated according to the following formula: risk score = (*β* mRNA1∗expression of mRNA1) + (*β* mRNA2∗expression of mRNA2) + ⋯+(*β* mRNAn∗expression of mRNAn). Based on the median risk score, patients with survival data were divided into high- and low-risk groups, and then, the association of risk score with survival and clinical features of patients was investigated by Kaplan–Meier survival analysis, which further evaluate the predictive ability of the prognostic signature. The results were validated in the ICGC and GSE57495 datasets.

### 2.4. Analyses of Clinical Significance and Prognostic Value of the Gene Signature in Pancreatic Cancer

The associations of the hypoxia-related prognostic gene signature with the clinical features in pancreatic cancer were first analyzed in the TCGA-PAAD dataset and then validated in the ICGC-PACA dataset. A two-tailed *P* value of less than 0.05 was considered statistically significant. The prognostic value of the signature in predicting the 1-, 3-, and 5-year survival probability of pancreatic cancer patients was determined using time-dependent receiver operating characteristic (time-ROC) curves, and the results were estimated by the area under the curve (AUC), which was running in R packages of “timeROC” [17].

### 2.5. Estimation of Immune Cell-Type Fractions in Pancreatic Cancer Tissues

To investigate the association of the gene signature with immune cells in the context of pancreatic cancer, the mRNA expression matrix of the TCGA-PAAD, ICGC-PACA, and GSE57495 datasets was normalized and analyzed using the CIBERSORT tool, which was used to estimate the fractions of 22 human immune cells, and the results were visualized using a Heatmap graph. Then, the differences in each immune cell fraction between the high- and low-risk groups were determined. *P* < 0.01 was considered statistically significant.

### 2.6. Gene Function and Pathway Analysis

The “clusterProfiler” package [18] was used to perform the gene function (Biological Process, BP) and KEGG pathway analysis for the hypoxia-related DEGs. Gene set enrichment analysis (GSEA) was conducted to evaluate the signaling pathways regulated by the genes and the hypoxia-related signature, with *P* < 0.01 considered significant pathways.

### 2.7. Association of DNA Methylation with Gene Expression

The DNA methylation data of each gene of the signature in pancreatic cancer were retrieved from the TCGA database, and the methylation levels of genes between pancreatic cancer tissues and normal tissues were compared. The correlations between methylation levels and mRNA expression were analyzed. The survival time between high- and low-methylation levels of each gene was analyzed using the Kaplan–Meier (KM) curve and the log-rank method. *P* < 0.01 was considered statistically significant. All these analyses were conducted using the DNA Methylation Interactive Visualization Database [19] (DNMIVD, http://119.3.41.228/dnmivd/index/).

### 2.8. Validation of the Protein Expression of Genes in the Human Protein Atlas Database

To further explore the protein expression of genes in the signature in pancreatic cancer and normal tissues, immunohistochemistry staining of these genes was performed using the Human Protein Atlas database (http://www.proteinatlas.org).

## 3. Results

### 3.1. Identification of Hypoxia-Related DEGs in Pancreatic Cancer and Analysis of their Function

**Figure 1:**
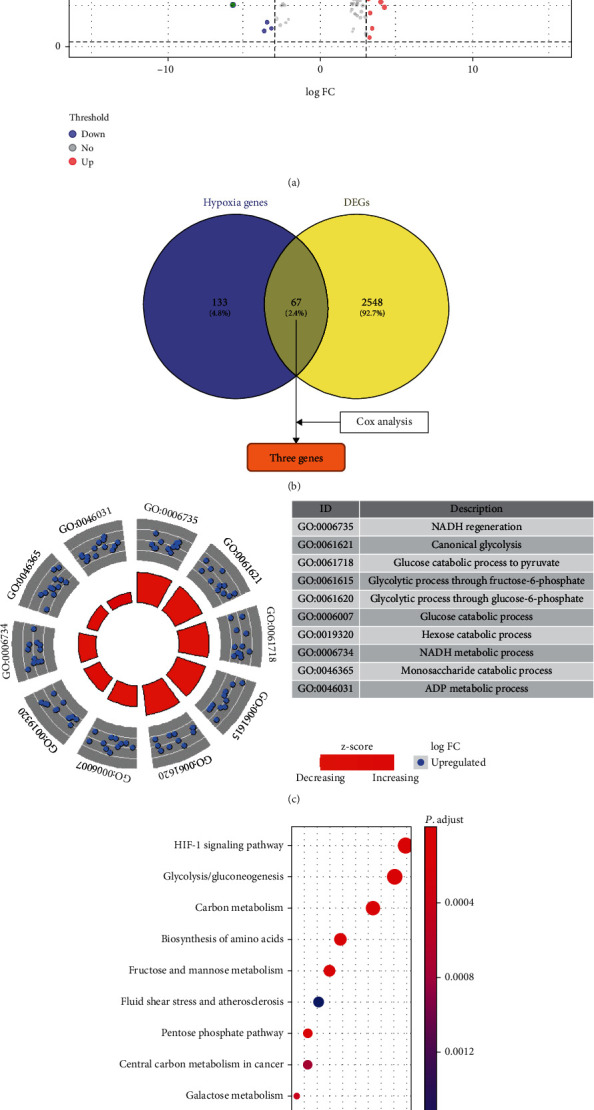
(a) Volcano plot of 2615 differentially expressed genes (DEGs) between pancreatic cancer and noncancerous pancreatic tissues; (b) 67 overlapped genes between DEGs and hypoxia induced genes. (c) Biology process enrichment of 67 overlapped genes; (d) KEGG pathway enrichment of 67 overlapped genes.

We first screened DEGs using the gene profile of pancreatic cancer from the TCGA database based on the selection criteria, and 2615 DEGs were identified between pancreatic cancer and adjacent noncancerous tissues (Table S2, Figure 1(a)). We then overlapped the DEGs with the hypoxia gene markers and obtained 67 hypoxia-related DEGs in pancreatic cancer (Figure 1(b)). Gene ontology enrichment and KEGG analysis revealed that these genes were enriched in NADH regeneration, canonical glycolysis, and glucose catabolism to pyruvate (Figure 1(c)). The most enriched pathways were the HIF-1 signaling pathway, glycolysis, gluconeogenesis, and carbon metabolism (Figure 1(d)).

### 3.2. Construction and Validation of a Hypoxia-Related Signature in Pancreatic Cancer

**Figure 2:**
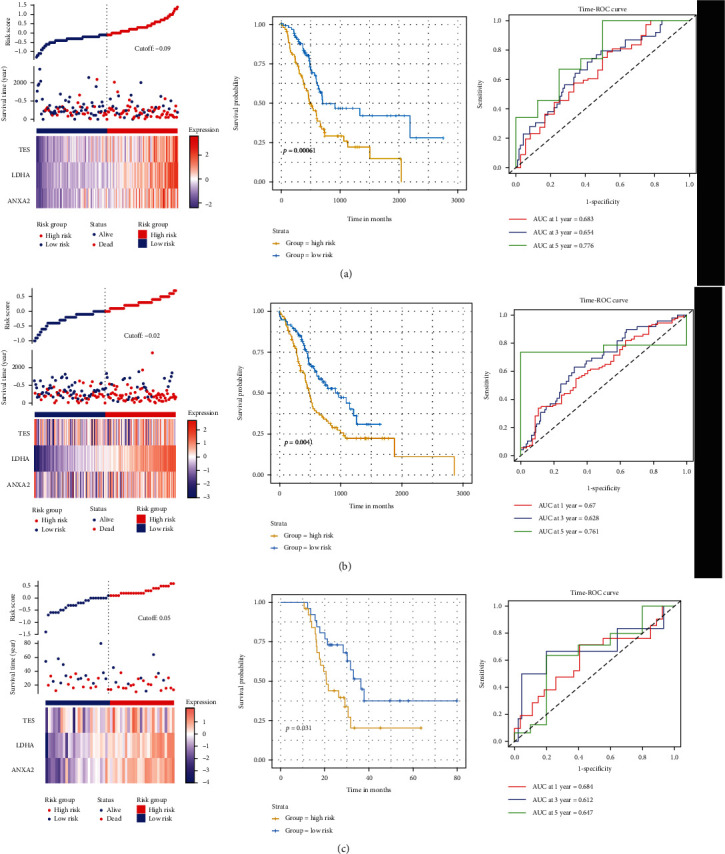
Construction and validation of three-hypoxia-related signature in pancreatic cancer. (a) Pancreatic cancer data from TCGA database; (b) pancreatic cancer data from ICGC database; (c) pancreatic cancer data from GSE57495 dataset.

First, we screened the hypoxia-related genes that associated with the prognosis of pancreatic cancer using the univariate Cox regression by analyzing the 67 hypoxia-related DEGs and identified three DEGs (TES, LDHA, and ANXA2) conforming to the proportional hazards assumption were significantly related to the OS of patients with pancreatic cancer (*P* < 0.01). Then, the coefficients of these three genes from multivariate Cox regression were multiplied by their expression to establish a prognostic signature, which is calculated as follows: risk score = (1.251∗TES expression) + (1.398∗LDHA expression) + (0.373∗ANXA2 expression). Using the median risk score as the cutoff value, the patients were divided into high- and low-risk groups. The KM curve indicated that the OS of patients in the high-risk group was significantly shorter than that of the low-risk group (*P* < 0.05). The area under the time-dependent ROC curves (AUCs) for 1-, 3-, and 5-year OS were 0.683, 0.654, and 0.776, respectively (Figure 2(a)), indicating a good predictive performance of this prognostic model.

To validate the prognostic value of the signature, the pancreatic cancer datasets of ICGC-PACA and GSE57495 datasets were used. Similar to the above results, the results from ICGC-PACA dataset revealed that, after dividing patients into the high- and low-risk groups with a median risk score as cutoff, the patients in the high-risk group had poorer survival than those in the low-risk group (*P* = 0.004). The AUCs of the prognostic model were 0.670, 0.628, and 0.761 for the 1-, 3-, and 5- year survival times, respectively (Figure 2(b)). In addition, the results from the GSE57495 dataset indicated that the patients in the high-risk group also had poorer survival than the low-risk group (*P* = 0.031). The AUCs of the prognostic model were 0.684, 0.612, and 0.647 for the 1-, 3-, and 5- year survival times, respectively (Figure 2(c)). Taken together, these results suggest a reliable and high prognostic value of the three-hypoxia-related signature in pancreatic cancer.

### 3.3. Hypoxia-Related Signature Is Correlated with Clinical Features in Pancreatic Cancer

**Figure 3:**
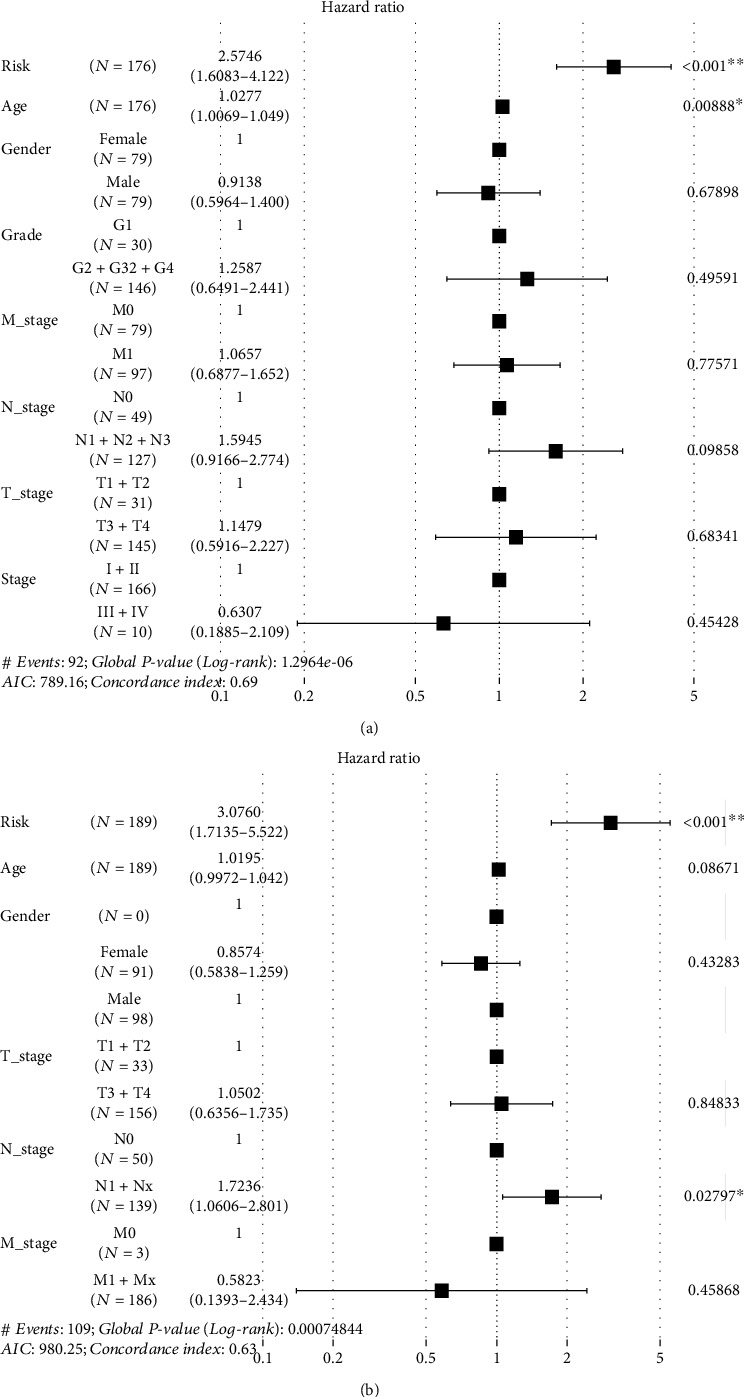
Prognostic value of hypoxia-related signature in pancreatic cancer. (a) Analysis of TCGA-PAAD dataset; (b) analysis of ICGC-PACA dataset.

To explore the clinical significance of the three hypoxia-related gene signatures in pancreatic cancer, we analyzed the association of the risk score of the signature with the clinical features in pancreatic cancer from the TCGA-PAAD and ICGC-PACA datasets. As shown in Table 1, the risk score of the signature was related to the grade and T stage of pancreatic cancer (*P* < 0.05), but not to the age, gender, N stage, and M stage of patients in the TCGA dataset (*P* < 0.05). Similar to the results of the TCGA dataset, the results from the ICGC dataset indicated that the risk score of signatures was related to the grade of cancer (*P* < 0.05), but not to age, gender, and TNM stage (*P* > 0.05). To compare the prognostic value of the gene signature with other clinical features in predicting the prognosis, we analyzed the survival data from the TCGA dataset and the ICGC dataset, and the results showed that only the risk score of signatures was significantly associated with the prognosis in patients with pancreatic cancer (*P* < 0.05; Figure 3).

### 3.4. Immune Cells Associated with the Hypoxia-Related Signature

**Figure 4:**
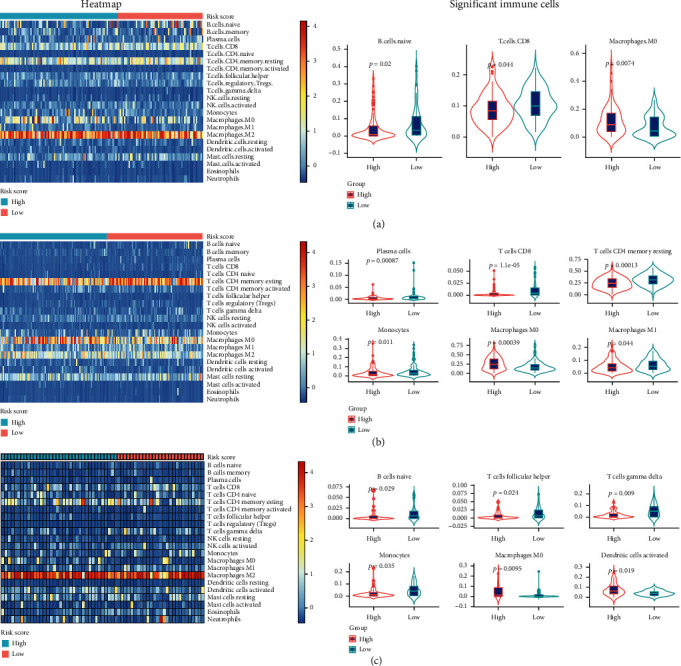
Landscape and significant proportion of 22 immune cells between high- and low-risk score of signatures in (a) TCGA-PAAD datasets, (b) ICGC-PACA datasets, and (c) GSE57495 datasets.

The effect of hypoxia on the immune microenvironment is crucial to the pathogenesis and progression of cancers [20, 21]. To examine the association of the hypoxia signature with the immune microenvironment in pancreatic cancer, we estimated the immune infiltration of 22 immune cell types using the CIBERSORT method in TCGA-PAAD, ICGC-PACA, and GSE57495 datasets, respectively (Figure 4). We depicted the landscape of 22 immune cell types in pancreatic cancer by dividing them into the low- and high-risk groups and found that the proportion of most immune cells was relatively small in pancreatic cancer. Only the macrophages and T cells accounted for a larger proportion than other immune cells in the three datasets did. When comparing the risk score in each immune cell, we found that the proportions of B cells, T cells, and macrophages were significantly different between the high- and low-risk groups (*P* < 0.05). Furthermore, when analyzing the correlations between three hypoxia gene expression and immune cell expression in pancreatic cancer, we found that the correlations were varied among the three datasets (TCGA-PAAD, ICGC-PACA, and GSE57495), but we also noted that only the Macrophages M0 significantly correlated to the three genes among the three datasets (*P* < 0.05, Table 2).

### 3.5. Effect of Methylation on the Expression of Hypoxia-Related Genes

**Figure 5:**
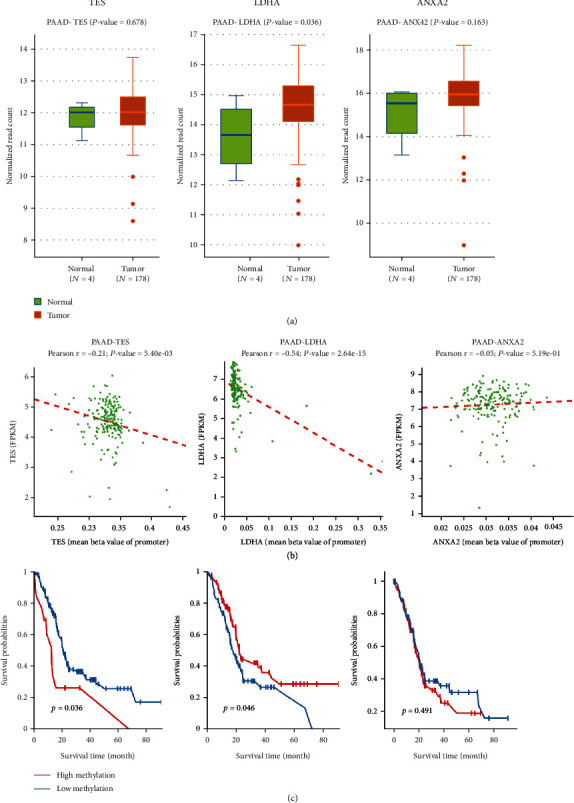
(a) Expression of methylation in pancreatic cancer and normal tissues; (b) correlation between methylation levels and mRNA in pancreatic cancer; (c) survival analysis of TES, LDHA, and ANXA2 in patients with pancreatic cancer.

Since these three hypoxia-related genes were differentially expressed in pancreatic cancer, and the methylation of DNA has been confirmed to affect the expression of genes, we further explored the methylation levels of each gene. Using the methylation data of pancreatic cancer from the TCGA database, we found that only LDHA methylation was significantly higher in pancreatic cancer tissues than in normal pancreatic tissues (*P* = 0.029), while no significant differences were found in TES and ANXA2 (*P* > 0.05). The correlation analysis revealed that the mRNA expression of TES and LDHA was negatively correlated with their methylation levels (*P* < 0.05). Furthermore, we also found that the expression of TES and LDHA was significantly associated with survival of pancreatic cancer patients (Figure 5). These results uncovered the effect of methylation on the regulation of hypoxia-related genes.

### 3.6. Validation of TES, LDHA, and ANXA2 Protein Expression in Pancreatic Tissues

**Figure 6:**
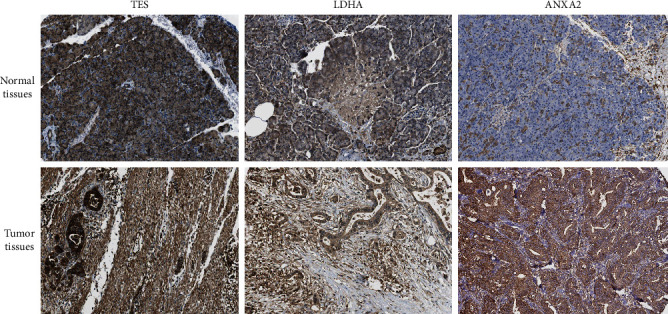
Representative immunohistochemistry staining of TES, LDHA, and ANXA2 in pancreatic normal tissues and cancer tissues derived from the HPA database.

Based on the immunohistochemical staining results of the pancreas from the Human Protein Atlas database, we found that the protein expression of TES, LDHA, and ANXA2 was not expressed in normal pancreatic tissues, whereas medium and high expression levels of these genes were observed in pancreatic cancer tissues (Figure 6), which was in agreement with the mRNA results from the TCGA-PAAD dataset. In summary, these results indicated that the mRNA and protein expression levels of the three genes were overexpressed in pancreatic cancer.

### 3.7. GSEA Identified Pathways That Were Regulated by TES, LDHA, and ANXA2

**Figure 7:**
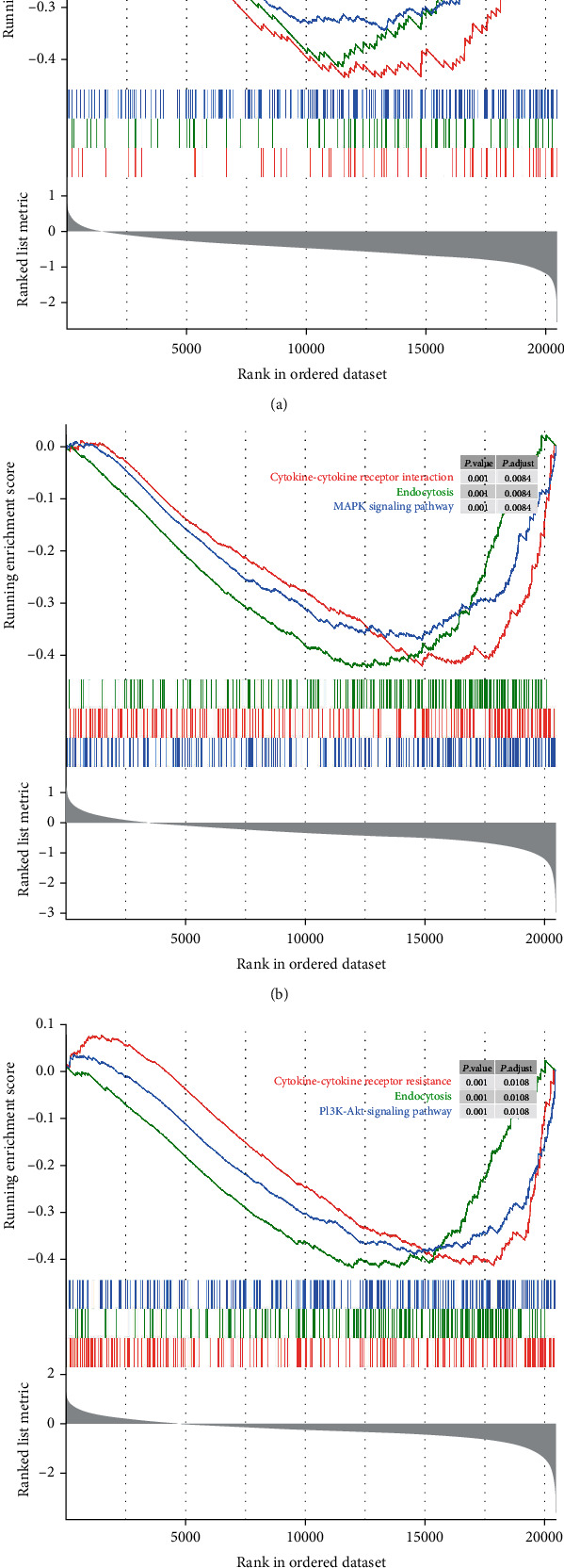
GSEA identified the pathways that TES, LDHA, and ANXA2 involved. (a) Pathways of TES; (b) pathways of LDHA; and (c) pathways of ANXA2.

To examine the pathways that TES, LDHA, and ANXA2 were involved, we performed GSEA using the TCGA-PAAD dataset. As shown in Figure 7, TES was mainly involved in EGFR tyrosine kinase inhibitor resistance (*P* < 0.001), and LDHA and ANXA2 were both involved in cytokine-cytokine receptor interaction pathways (*P* < 0.001).

## 4. Discussion

In the present study, we identified three hypoxia-related genes that are associated with the prognosis of patients with pancreatic cancer using the gene profile and established a prognostic signature using the above three genes. The prognostic value of this signature was validated by two independent cohorts, and the results further guarantee the robustness of the prognostic value. Next, we examined the association of the signature with the clinical features, pathways, and the immune microenvironment. Finally, we explored the effect of DNA methylation on gene expression, and the pathways regulated by these three genes, and confirmed the protein expression of these genes. Considering the widely varying prognostic outcomes of pancreatic cancer, our results provide a relatively reliable signature to clarify patients with different risks and prognoses, which greatly help in the selection of personalized strategy and timely follow-up for high-risk patients.

Hypoxia is a common feature that occurs during the development of solid tumors. Evidence has shown hypoxia to be associated with poor prognosis in patients with several cancers. Targeting hypoxia might be an effective strategy to overcome hypoxia-associated resistance in cancer treatment [22, 23]. Currently, precisely predicting the prognosis of patients with pancreatic cancer remains a great challenge [24]. Since hypoxia plays critical roles in the progression of pancreatic cancer, many studies have employed hypoxia-related genes to predict the survival of these patients, finding that these genes have a good prognostic performance [11–13]. For example, HIF-1*α* has been widely studied in many cancers, including pancreatic cancer, and overexpression of HIF-1*α* is significantly associated with poor prognosis in pancreatic cancer [25]. Wang et al. [26] found that overexpression of STIM1 mediated by HIF-1*α* promotes pancreatic cancer progression and STIM1 is a potential prognostic marker for treatment.

In addition to the single hypoxia-related gene, the gene signature of several genes has been studied in several cancers, such as lung cancer [27] and gastric cancers [28], which could achieve a more reliable prognostic value than the single one. In the present study, we also analyzed the function and pathways of hypoxia-related DEGs and identified three hypoxia-related genes that were significantly associated with prognosis in pancreatic cancer patients. Our results demonstrated that the three hypoxia-related gene signatures could effectively predict the prognosis of patients with pancreatic cancer. Using two independent cohorts, the prognostic value of the signature was further validated. Interestingly, we found that this signature was associated only with the grade of pancreatic cancer, but not with other clinical features. The reasons for this are unknown, and further study is warranted.

The number and activity of immune cells in the TME are crucial to tumor pathogenesis and progression. Several immune cells have been reported to be associated with the prognosis and treatment effect of pancreatic cancer [29]. Recently, the relationship of the TME has been studied. Current evidence indicates that hypoxia could incapacitate immune effector cells [30] and enhance the activity of immunosuppressive cells [31], immune escape [32], and tumor cell adaptations to hypoxia [33], subsequently facilitating cancer cell invasion and metastasis. In recent years, immune cell strategies, such as CAR-T technology, have made great success in hematological malignances; however, their effect in solid tumors is unsatisfactory. This may be attributed to the fewer immune cells that undergo infiltration into solid tumors [34, 35], especially pancreatic cancer. In this study, we found that most of the 22 immune cells showed little infiltration in pancreatic cancer; only T cells and macrophages showed relatively higher infiltration. Our results also found that only macrophage M0 cells were significantly different between high- and low-risk signatures, suggesting that macrophage M0 cells might be closely related to the signature, but the mechanism needs to be further investigated.

Among the three hypoxia-related genes, the TES gene appeared to possess the properties of a tumor suppressor. Mutation analysis of the coding TES exons in 21 human tumor-derived cell lines revealed the presence of a frameshift mutation in one allele in the breast cancer cell line ZR-75 [36], but its role in pancreatic cancer has not been reported. Unlike TES, LDHA together with KLF4 [37] or c-Myc [38] has been found to positively regulate aerobic glycolysis and promote tumor progression in pancreatic cancer. A previous study showed that FOXM1 promotes the Warburg effect and pancreatic cancer progression via transactivation of LDHA expression [39]. Regarding ANXA2, high expression of ANXA2 is associated with DNA repair, metabolic alteration, and worse survival in pancreatic cancer [40] Moreover, our results also revealed their methylation levels with gene expression and patient survival, and the pathways they involved in, which provided more information about their role in pancreatic cancer. However, no previous study has investigated the role of these genes in cancers under hypoxia conditions.

There are a number of articles regarding a type of gene that can be used as a prognostic marker for human tumors. A recent study used a 4-gene-based hypoxia signature to predict the prognosis of pancreatic cancer patients [41]. Our study used the used two larger pancreatic cancer sets from ICGC and GEO database, which guaranteed the robustness of our results. In addition, we analyzed the DNA methylation with gene expression and validated the gene expression at protein levels, which were not reported previously, the results provide novel insight into the role of these genes in the pathogenesis of pancreatic cancer.

However, several limitations of our study need to be noted. First, although we used two external independent cohorts to verify the results, the robustness needs to be further validated in other large cohorts. Second, the expression of hypoxia-related genes was tested by RNA sequencing or microarray. Other methods, such as RT-PCR, are warranted to test gene expression and verify our results. Third, immune cell infiltration was tested using gene markers, and flow cytometry should be used to determine the exact number of immune cells. Fourth, due to lack of clinical samples, we could not verify the expression of the three hypoxia-related genes in pancreatic cancer using clinical samples. Fifth, hypoxia-related genes were not reported in their role in pancreatic cancer under hypoxia condition. Therefore, *in vivo* and *in vitro* basic experiments of the three genes under hypoxia condition are needed to further validate their role in pathogenesis and progression of pancreatic cancer. Therefore, future study should be conducted to address the abovementioned limitations.

## 5. Conclusions

In conclusion, this study constructed a prognostic signature of three hypoxia-related genes, which is significantly associated with the prognosis of patients with pancreatic cancer. This signature could identify high infiltration of immune cells and has a better prognostic value than other clinical features. Our results provide potential translational value for the clinical management of patients with pancreatic cancer. Our results also shed light on the role of hypoxia-related genes in the pathogenesis and progression of pancreatic cancer and provide potential therapeutic targets.

## Figures and Tables

**Table 1:** Association of risk score of signature with clinical features in pancreatic cancer.

	TCGA dataset		ICGC dataset	
	Risk score	*P* value	Risk score	*P* value
Age				
>60 years	11.314 ± 0.707	0.144	10.719 ± 0.345	0.456
<60 years	11.467 ± 0.621		10.767 ± 0.395	
Gender				
Female	11.381 ± 0.668	0.773	10.737 ± 0.358	0.919
Male	11.351 ± 0.696		10.732 ± 0.364	
Grade				
G1	10.765 ± 1.15	0.002	10.749 ± 0.358	0.016
*G*2 + *G*32 + *G*4	11.487 ± 0.455		10.444 ± 0.298	
T stage				
*T*1 + *T*2	10.919 ± 1.138	0.014	10.788 ± 0.283	0.264
*T*3 + *T*4	11.459 ± 0.493		10.723 ± 0.374	
N stage				
N0	11.22 ± 0.903	0.153	10.741 ± 0.352	0.889
*N*1 + *N*2 + *Nx*	11.42 ± 0.569		10.732 ± 0.364	
M stage				
M0	11.457 ± 0.485	0.088	10.548 ± 0.158	0.165
M1 + Mx	11.289 ± 0.802		10.738 ± 0.362	
Clinical stage				
I + II	11.374 ± 0.667	0.571		
III + IV	11.2 ± 0.926			
Chemotherapy				
Yes	11.29 ± 0.79	0.276		
No	11.42 ± 0.61			
Radiation				
Yes	11.39 ± 0.59	0.941		
No	11.38 ± 0.70			

**Table 2:** Correlation of ANXA2, LDHA and TES with immune cells.

	TCGA-PAAD			ICGC-PACA			GSE57495		
	ANXA2	LDHA	TES	ANXA2	LDHA	TES	ANXA2	LDHA	TES
B cells memory	0.387	0.461	0.642	0.038	0.017	0.516	0.471	0.825	0.201
B cells naive	0.103	0.192	0.744	0.060	0.042	0.982	0.139	0.296	0.171
Dendritic cells activated	0.553	0.029	0.452	0.125	0.068	0.733	0.028	0.136	0.085
Dendritic cells resting	0.489	0.486	0.034	0.471	0.774	0.612	0.051	0.041	0.004
Eosinophils	0.557	0.181	0.789	0.979	0.193	0.726	0.568	0.017	0.193
Macrophages M0	0.000	0.000	0.031	0.016	0.000	0.472	0.002	0.022	0.010
Macrophages M1	0.157	0.243	0.378	0.358	0.023	0.754	0.036	0.281	0.934
Macrophages M2	0.362	0.514	0.823	0.593	0.184	0.362	0.579	0.670	0.526
Mast cells activated	0.252	0.694	0.019	0.453	0.201	0.495	0.666	0.703	0.627
Mast cells resting	0.121	0.235	0.867	0.132	0.514	0.210	0.305	0.856	0.514
Monocytes	0.072	0.044	0.605	0.240	0.061	0.232	0.114	0.156	0.014
Neutrophils	0.294	0.008	0.252	0.696	0.228	0.848	0.162	0.025	0.083
NK cells activated	0.773	0.197	0.068	0.856	0.151	0.698	0.109	0.188	0.623
NK cells resting	0.901	0.204	0.712	0.053	0.357	0.988	0.237	0.209	0.352
Plasma cells	0.143	0.274	0.635	0.095	0.042	0.387	0.107	0.153	0.929
T cells CD4 memory activated	0.318	0.837	0.916	0.303	0.359	0.945	0.728	0.187	0.252
T cells CD4 memory resting	0.002	0.041	0.727	0.014	0.000	0.731	0.727	0.150	0.479
T cells CD4 naive	0.920	0.400	0.868	0.219	0.360	0.255	0.375	0.978	0.832
T cells CD8	0.029	0.001	0.070	0.000	0.000	0.347	0.209	0.136	0.888
T cells follicular helper	0.330	0.339	0.050	0.968	0.006	0.259	0.000	0.052	0.072
T cells gamma delta	0.237	0.579	0.329	0.022	0.020	0.214	0.016	0.022	0.001
T cells regulatory Tregs	0.007	0.140	0.926	0.005	0.904	0.916	0.617	0.641	0.497

PAAD: pancreatic cancer; PACA: pancreatic adenocarcinoma.

## Data Availability

The data that support the findings of this study are openly available in The Cancer Genome Atlas (TCGA) data portal, Gene Expression Omnibus (GEO) database.

## Supplementary Materials

Supplementary Materials Table S1. The hallmark gene sets of hypoxia were retrieved from Molecular Signatures Database. Table S2. Differentially expressed genes were identified between pancreatic cancer and adjacent noncancerous tissues in TCGA-PAAD dataset.
