# Supplementary material for: Hypoxia-Related Signature Is a Prognostic Biomarker of Pancreatic Cancer
Source: Dis Markers. 2022 Jun 25;2022:6449997. doi: 10.1155/2022/6449997 (PMC9250441; doi:10.1155/2022/6449997)
Supplement: Supplementary Materials — Table S1. The hallmark gene sets of hypoxia were retrieved from Molecular Signatures Database. Table S2. Differentially expressed genes were identified between pancreatic cancer and adjacent noncancerous tissues in TCGA-PAAD dataset. [file 6449997.f1.pdf]

HALLMARK_HYPOXIA
> Genes up-regulated in response to low oxygen levels (hypoxia).

ACKR3
ADM
ADORA2B
AK4
AKAP12
ALDOA
ALDOB
ALDOC
AMPD3
ANGPTL4
ANKZF1
ANXA2
ATF3
ATP7A
B3GALT6
B4GALNT2
BCAN
BCL2
BGN
BHLHE40
BNIP3L
BRS3
BTG1
CA12
CASP6
CAV1
CAVIN1
CAVIN3
CCN1
CCN2
CCN5
CCNG2
CDKN1A
CDKN1B
CDKN1C
CHST2
CHST3
CITED2
COL5A1
CP
CSRP2
CXCR4
DCN
DDIT3
DDIT4

DPYSL4
DTNA
DUSP1
EDN2
EFNA1
EFNA3
EGFR
ENO1
ENO2
ENO3
ERO1A
ERRFI1
ETS1
EXT1
F3
FAM162A
FBP1
FOS
FOSL2
FOXO3
GAA
GALK1
GAPDH
GAPDHS
GBE1
GCK
GCNT2
GLRX
GPC1
GPC3
GPC4
GPI
GRHPR
GYS1
HAS1
HDLBP
HEXA
HK1
HK2
HMOX1
HOXB9
HS3ST1
HSPA5
IDS
IER3
IGFBP1
IGFBP3

IL6
ILVBL
INHA
IRS2
ISG20
JMJD6
JUN
KDELR3
KDM3A
KIF5A
KLF6
KLF7
KLHL24
LALBA
LARGE1
LDHA
LDHC
LOX
LXN
MAFF
MAP3K1
MIF
MT1E
MT2A
MXI1
MYH9
NAGK
NCAN
NDRG1
NDST1
NDST2
NEDD4L
NFIL3
NOCT
NR3C1
P4HA1
P4HA2
PAM
PCK1
PDGFB
PDK1
PDK3
PFKFB3
PFKL
PFKP
PGAM2
PGF

PGK1
PGM1
PGM2
PHKG1
PIM1
PKLR
PKP1
PLAC8
PLAUR
PLIN2
PNRC1
PPARGC1A
PPFIA4
PPP1R15A
PPP1R3C
PRDX5
PRKCA
PYGM
RBPJ
RORA
RRAGD
S100A4
SAP30
SCARB1
SDC2
SDC3
SDC4
SELENBP1
SERPINE1
SIAH2
SLC25A1
SLC2A1
SLC2A3
SLC2A5
SLC37A4
SLC6A6
SRPX
STBD1
STC1
STC2
SULT2B1
TES
TGFB3
TGFBI
TGM2
TIPARP
TKTL1

TMEM45A
TNFAIP3
TPBG
TPD52
TPI1
TPST2
UGP2
VEGFA
VHL
VLDLR
WSB1
XPNPEP1
ZFP36
ZNF292

Symbol	Mean.Tumor	Mean.Normal	Log2FC	adj.pval
A2M	183.787	28.3	2.657	4.39E-74
A4GALT	17.14	2.2	2.503	5.80E-72
ABCG1	16.66	1.21	2.998	5.46E-113
ABHD11-AS1	10.2	1.07	2.436	9.64E-55
ABHD17C	35.111	2.48	3.375	8.09E-110
ABI2	15.47	3.1	2.006	1.90E-89
ABI3	10.11	1.39	2.217	9.18E-70
ABLIM3	15.77	1.15	2.963	6.07E-96
ABR	41.92	9.11	2.086	1.35E-81
ABRACL	45.351	5.66	2.799	1.73E-95
AC005152	0.32	4.91	-2.163	4.87E-61
AC005255	5.78	0	2.761	1.23E-97
AC005339	5.63	0.47	2.173	2.33E-81
AC005786	8.19	0.26	2.867	4.28E-48
AC005943	4.19	0	2.376	2.47E-29
AC005943	5.18	0	2.628	1.74E-18
AC005944	19.401	2.32	2.619	1.28E-61
AC007283	5.52	0	2.705	2.17E-101
AC009065	3.66	0	2.22	6.42E-118
AC011298	0.16	15.39	-3.821	1.58E-124
AC011558	104.27	13.79	2.831	2.79E-56
AC021066	129.48	6.07	4.206	1.03E-24
AC078941	0.93	22.95	-3.633	3.18E-88
AC093642	0.73	12.44	-2.958	8.78E-95
AC093673	11.83	1.03	2.66	8.55E-108
AC093850	5.16	0	2.623	1.15E-65
AC131097	0.19	12.14	-3.465	1.07E-88
AC132217	28.779	4.74	2.375	1.33E-26
AC156455	2.03	11.43	-2.036	4.57E-77
AC239868	26.06	0.25	4.436	2.47E-64
AC239868	26.06	0.25	4.436	2.47E-64
ACADL	0.66	16.959	-3.435	6.15E-127
ACE	19.311	1.95	2.783	3.19E-112
ACOT7	25.189	3.51	2.538	6.54E-114
ACP5	55.479	5.22	3.183	2.50E-75
ACSL3	38.869	6.61	2.389	1.17E-108
ACSL4	17.629	2.54	2.396	3.16E-91
ACSL5	45.251	1.71	4.093	5.15E-106
ACTA2	540.517	26.339	4.308	4.33E-91
ACTB	4048.578	313.147	3.688	6.57E-103
ACTG2	16.64	2.09	2.513	1.02E-42
ACTN1	151.355	22.529	2.695	2.24E-88
ACTN4	302.983	49.07	2.602	1.01E-86
ACTR2	83.562	11.58	2.749	8.13E-107
ACTR3	105.36	14.8	2.751	3.04E-90
ACVR1	25.81	3.66	2.524	2.57E-105

ACY1	40.14	7.21	2.325	5.52E-84
ADAM10	41.599	6.66	2.475	4.54E-80
ADAM12	5.1	0.05	2.538	1.30E-64
ADAM15	106.957	22.6	2.194	8.03E-78
ADAM19	8.42	0.24	2.925	2.72E-89
ADAM28	29.67	1.84	3.433	2.90E-80
ADAM8	28.889	2.46	3.111	4.54E-83
ADAM9	71.322	5.26	3.53	6.43E-98
ADAMTS12	6.53	0.18	2.674	1.54E-80
ADAMTS2	22.409	2.05	2.94	1.08E-82
ADAP1	43.761	5.76	2.727	1.05E-53
ADCY7	8.25	1.01	2.202	3.82E-67
ADGRA2	17.759	1.7	2.797	7.77E-78
ADGRE5	62.769	12.35	2.256	1.42E-71
ADGRF1	11.63	0.08	3.548	2.68E-86
ADGRG1	92.308	21.85	2.03	1.69E-69
ADGRG6	8.16	0.79	2.355	2.57E-74
ADH1C	20.129	4.16	2.034	9.98E-24
ADHFE1	3.18	16.549	-2.07	4.08E-86
ADTRP	3.85	0.1	2.14	1.05E-67
AEBP1	274.24	12.62	4.337	4.82E-94
AF131216	0.05	6.32	-2.801	3.46E-81
AFAP1	13.4	1.19	2.717	1.10E-110
AFAP1-AS1	4.46	0.02	2.42	2.87E-62
AGR2	438.276	4.23	6.392	5.17E-107
AGR3	67.878	1.67	4.689	9.31E-88
AGT	16.05	2.23	2.4	5.36E-43
AHNAK	87.262	11.45	2.826	9.16E-89
AHNAK2	40.509	0.51	4.781	5.35E-103
AHR	32.32	2.3	3.336	9.13E-108
AIF1	59.218	3.67	3.689	4.53E-86
AIG1	29.871	6.48	2.045	7.21E-103
AIM1L	9	0.16	3.108	8.01E-86
AK1	80.359	14.47	2.395	7.91E-95
AK4	8.98	0.31	2.929	1.07E-92
AKAP12	14.8	1.72	2.538	2.50E-61
AKAP2	16.57	2.05	2.526	6.22E-80
AKR1B10	22.641	0.24	4.253	1.33E-66
AKR1C2	17.2	3.34	2.068	7.46E-38
AKR1C3	79.688	14.57	2.374	1.55E-56
AKR7A3	17.34	104.69	-2.527	6.95E-26
AL442127	0	6.32	-2.872	6.42E-18
AL513523	17.49	3.13	2.163	8.63E-61
ALAS1	37.53	6.31	2.398	1.24E-113
ALB	4.14	255.857	-5.643	1.44E-86
ALDH1A3	18.69	3.03	2.289	1.47E-49
ALDH1B1	15.64	2.99	2.06	3.22E-80

ALDH2	139.478	22.841	2.559	3.56E-87
ALDH3A1	6.56	0.17	2.692	9.17E-59
ALDH3B1	35.089	2.88	3.217	1.35E-72
ALDOA	1162.084	235.763	2.296	7.44E-71
ALDOC	15.17	1.67	2.598	2.75E-91
ALOX5AP	63.579	4.64	3.517	1.12E-72
ALYREF	60.732	11.86	2.263	1.24E-106
AMICA1	12.89	0.95	2.833	9.38E-66
AMIGO2	17.64	1.37	2.975	1.56E-94
AMN	13.17	0.38	3.36	1.99E-66
AMY2A	4.86	8429.9	-10.491	2.21E-57
AMY2B	11.68	1523.738	-6.91	1.80E-68
AMYP1	0.13	11.61	-3.48	9.17E-16
ANGPTL2	23.88	1.55	3.286	6.08E-94
ANKH	53.16	9.39	2.382	2.68E-87
ANLN	7.22	0.17	2.813	4.13E-100
ANO1	50.251	1.53	4.34	5.01E-117
ANP32A	97.342	20.74	2.177	1.48E-93
ANP32E	19.74	3.1	2.339	8.11E-113
ANPEP	38.289	306.469	-2.968	8.54E-49
ANTXR1	51.911	1.34	4.499	1.09E-105
ANTXR2	44.27	3.62	3.293	2.60E-80
ANXA1	250.678	11.12	4.376	1.24E-99
ANXA10	55.529	5.31	3.163	1.12E-29
ANXA11	270.614	53.319	2.322	1.59E-81
ANXA13	5.76	0.08	2.646	3.62E-60
ANXA2	1875.942	115.151	4.014	4.22E-98
ANXA2P2	30.64	1.17	3.866	2.57E-120
ANXA3	93.798	13.43	2.716	1.03E-52
ANXA5	370.954	69.189	2.406	2.30E-81
ANXA8	12.49	0.1	3.616	2.52E-66
ANXA8L1	9.11	0.11	3.187	2.63E-62
AOC1	32.17	0.1	4.914	2.12E-113
AOC3	21.44	3.12	2.445	4.79E-61
AOX1	3.92	33.721	-2.819	5.39E-65
AP000349	63.548	0.01	5.998	2.64E-105
AP002518	109.273	0	6.785	1.75E-68
AP003391	8.62	0	3.266	2.59E-21
AP006621	0.54	11.28	-2.995	8.66E-97
AP1M2	78.738	18.25	2.05	5.90E-70
AP1S1	59.962	11.44	2.293	6.74E-114
AP1S3	10.13	0.74	2.677	1.23E-91
AP2B1	65.298	12.59	2.286	1.34E-104
AP2M1	245.962	51.631	2.23	3.96E-91
APCDD1	20.26	4.07	2.068	7.92E-58
APCS	17.25	1.37	2.945	7.04E-19
APLNR	11.99	0.46	3.153	1.03E-79

APLP2	251.409	38.321	2.682	1.12E-99
APOBEC1	3.1	0	2.036	9.04E-51
APOBEC3C	20.05	1.8	2.91	3.09E-101
APOBEC3G	10.56	0.91	2.598	4.93E-96
APOBR	6.16	0.38	2.375	3.97E-82
APOC1	213.53	3.58	5.55	4.75E-94
APOC2	6.95	0.16	2.777	2.72E-70
APOC4-APOC	10.69	0.36	3.104	8.22E-73
APOE	220.295	10.01	4.329	1.34E-79
APOL1	135.447	2.76	5.181	1.60E-128
APOL2	41.81	6.06	2.6	3.57E-94
APOL3	20.141	3.66	2.182	1.16E-66
APOL6	8.2	0.8	2.354	2.95E-102
APP	327.079	65.321	2.307	1.81E-83
AQP12A	0.46	69.18	-5.587	1.18E-111
AQP12B	0.31	161.791	-6.957	2.01E-125
AQP5	37.281	0.48	4.693	2.38E-76
AQP8	0.36	173.488	-7.003	4.01E-82
ARAP1-AS1	13.92	0	3.899	5.03E-67
AREG	22.259	0.6	3.862	2.83E-86
ARF3	69.84	14.39	2.203	1.29E-97
ARF6	48	6.32	2.743	3.86E-122
ARFIP1	21.48	4	2.169	4.59E-110
ARHGAP1	67.409	14.13	2.177	1.62E-90
ARHGAP15	6.06	0.52	2.216	1.26E-66
ARHGAP23	13.16	1.42	2.549	1.41E-78
ARHGAP25	6.29	0.8	2.018	3.10E-67
ARHGAP26	13.51	1.61	2.475	4.63E-83
ARHGAP27	31.871	4.13	2.68	2.31E-77
ARHGAP30	7.66	0.77	2.291	1.19E-67
ARHGDIB	162.883	10.77	3.799	2.29E-101
ARHGDIG	1.4	51.239	-4.444	1.53E-111
ARHGEF2	43.501	5.49	2.778	1.03E-99
ARID5B	8.27	1.04	2.184	8.72E-78
ARL14	21.681	2.59	2.659	4.72E-45
ARL15	11.76	1.83	2.173	3.07E-97
ARL4A	21.651	2.55	2.674	1.59E-119
ARL4C	34.539	1.67	3.735	1.90E-99
ARL4D	7.1	0.77	2.194	2.74E-72
ARL6IP1	140.088	17.55	2.927	2.07E-110
ARL6IP5	100.412	16.99	2.495	4.08E-97
ARL8A	47.29	10.71	2.044	1.16E-104
ARMC9	10.14	1.77	2.008	8.86E-90
ARNTL2	7.62	0.41	2.612	2.41E-101
ARPC1B	304.584	24.8	3.566	2.41E-112
ARPC2	286.799	41.24	2.768	5.94E-106
ARPC3	328.829	67.111	2.276	1.30E-91

ARPC4	132.43	26.67	2.27	1.85E-94
ARPC5	155.664	21.091	2.826	7.95E-114
ARPC5L	48.431	10.86	2.059	8.42E-103
ARRDC2	38.869	6.99	2.319	6.07E-83
ASAH1	92.25	21.69	2.039	2.56E-86
ASAP2	14.5	1.93	2.403	6.08E-78
ASF1B	7.33	0.22	2.771	2.43E-118
ASPH	75.462	12.14	2.541	1.78E-85
ASPHD1	21.739	3.93	2.206	2.92E-65
ASPHD2	5.25	0.2	2.381	1.88E-103
ASPN	38.361	1.25	4.129	2.10E-94
ASS1	163.369	19.329	3.015	3.47E-92
ATHL1	34.21	374.831	-3.416	1.09E-59
ATOX1	131.936	19.849	2.673	8.61E-120
ATP1B1	244.043	37.61	2.666	2.33E-83
ATP1B3	101.863	14.61	2.72	1.81E-97
ATP2B4	33.19	5.52	2.391	1.05E-73
ATP2C1	53.578	12.01	2.069	1.36E-86
ATP2C2	8.62	0.51	2.672	4.68E-73
ATP4A	0.01	3.18	-2.049	2.05E-62
ATP5E	121.548	16.57	2.802	2.33E-112
ATP5J2	469.993	104.343	2.161	5.46E-93
ATP5L	330.429	72.458	2.174	1.29E-100
ATP6AP2	91.569	13.11	2.714	3.06E-115
ATP6V0D1	90.471	20.75	2.072	1.53E-95
ATP6V0E1	302.082	66.491	2.167	2.23E-92
ATP6V1D	76.531	13.45	2.424	7.81E-113
ATP6V1E1	108.007	22.59	2.208	4.01E-103
ATP6V1F	197.17	33.261	2.532	4.70E-111
ATP6V1G1	120.3	26.541	2.139	3.75E-96
AURKA	6.36	0.43	2.364	1.17E-102
AURKB	7.44	0.25	2.755	9.38E-100
AVPI1	16.59	3.35	2.016	8.01E-90
AXL	21.01	1.47	3.156	7.71E-92
AZGP1	54.36	240.55	-2.125	1.19E-31
B2M	8387.353	568.217	3.881	3.82E-97
B3GALT4	13.73	1.42	2.606	1.78E-124
B3GNT3	50.92	1.11	4.621	9.05E-123
B3GNT5	11.38	1.5	2.308	2.40E-88
B3GNT9	9.33	1.14	2.271	1.45E-105
B4GALNT3	17.65	1.69	2.794	2.33E-88
B4GALT1	59.449	12.15	2.201	7.47E-60
B4GALT4	35.76	7.01	2.198	2.69E-103
B4GALT5	33.92	3.49	2.959	6.40E-107
BACE2	99.27	20.3	2.235	1.62E-52
BAG3	33.15	6.45	2.197	6.70E-79
BAIAP2	38.561	4.24	2.916	9.37E-118

BAIAP2L2	19.2	0.82	3.472	1.83E-85
BAK1	25.43	4.5	2.265	5.51E-100
BAMBI	10.3	1.79	2.018	6.31E-74
BASP1	23.96	0.92	3.7	2.21E-95
BATF	17.99	0.36	3.804	9.06E-114
BAX	132.439	21.13	2.592	4.52E-109
BCAR3	15.3	1.57	2.665	1.53E-112
BCAS1	21.31	0.25	4.158	4.51E-91
BCAS4	6.92	0.62	2.29	4.56E-95
BCL10	13.92	1.81	2.409	3.31E-104
BCL2A1	8.61	0.23	2.966	6.40E-76
BCL2L1	116.129	18.88	2.559	2.96E-99
BCL7C	31.851	6.18	2.194	3.21E-107
BCR	42.83	7.89	2.302	2.37E-110
BEGAIN	1.17	11.54	-2.531	3.06E-100
BGN	613.066	22.19	4.727	3.46E-103
BHLHA15	0.81	117.051	-6.027	2.44E-110
BHLHE40	141.778	18.84	2.847	6.73E-60
BHLHE41	20.11	2.34	2.66	1.35E-83
BICD1	8.87	0.78	2.471	1.02E-109
BID	33.15	3.43	2.947	7.38E-95
BIK	22.36	0.78	3.714	3.68E-113
BIRC3	30.099	2.44	3.176	9.82E-66
BIRC5	6.61	0.25	2.606	2.53E-68
BLACAT1	3.59	0.05	2.128	2.31E-73
BLCAP	54.59	10.89	2.225	1.30E-104
BLOC1S1	178.106	41.749	2.067	4.54E-97
BLVRA	44.829	8.77	2.23	9.63E-102
BMP1	24.21	4.78	2.125	1.33E-68
BMP4	26.339	1.22	3.622	1.70E-96
BMPR2	12.48	1.84	2.247	9.46E-102
BNIP3	12.03	128.871	-3.317	7.56E-72
BNIP3L	36.839	6.17	2.4	9.72E-98
BOLA2	14.89	2.22	2.303	2.63E-97
BOLA2B	109.008	15.75	2.715	1.37E-115
BPGM	15.82	2.89	2.112	7.04E-105
BPIFB1	3.52	0.01	2.162	1.07E-38
BPNT1	21.17	2.84	2.529	9.87E-121
BRSK2	1.13	47.851	-4.519	1.21E-119
BSG	499.208	106.122	2.223	1.37E-90
BST1	8.69	0.48	2.711	3.32E-68
BST2	184.055	8.16	4.337	4.08E-102
BTBD10	18.09	3.46	2.098	2.83E-110
BTBD6	23.45	4.65	2.114	2.76E-58
BTN3A3	12.33	2.22	2.05	1.82E-41
BUB1	3.74	0.08	2.134	2.83E-102
BUD31	99.511	21.47	2.161	8.49E-100

BX470102	4.69	0.09	2.384	7.30E-70
BZW1	83.049	12.65	2.622	9.96E-94
BZW1P2	11.07	1.5	2.271	3.86E-69
C10orf54	47.199	6.11	2.761	1.67E-91
C11orf98	78.738	18.67	2.019	1.76E-96
C12orf49	16.37	3.29	2.018	3.33E-97
C12orf75	92.918	13.32	2.713	5.29E-72
C14orf119	52.91	12.41	2.007	2.33E-103
C14orf80	11.05	1.83	2.09	1.05E-84
C15orf48	104.119	2.46	4.925	3.31E-98
C19orf33	483.24	1.9	7.383	8.56E-125
C1GALT1	18.11	2.05	2.647	2.99E-103
C1orf106	64.82	5.12	3.427	4.56E-68
C1orf116	27.23	3.85	2.541	1.22E-81
C1orf162	20.15	3.55	2.217	2.18E-51
C1orf21	23.779	4.84	2.085	1.21E-72
C1orf233	5.36	0.29	2.302	1.90E-101
C1orf54	22.161	1.42	3.259	2.69E-107
C1QA	182.48	10.28	4.024	1.99E-85
C1QB	181.634	14.66	3.544	9.35E-69
C1QC	136.711	10.38	3.597	7.37E-72
C1QTNF1	33.91	5.2	2.493	4.66E-80
C1QTNF3	16.549	2.76	2.223	1.77E-53
C1QTNF5	87.432	7.27	3.419	4.00E-98
C1QTNF6	14.37	2.03	2.343	4.42E-77
C1R	261.632	34.881	2.872	9.31E-63
C1S	242.139	35.971	2.717	5.08E-54
C2	21.919	0.78	3.687	5.27E-106
C20orf24	97.558	23.039	2.036	7.12E-96
C2orf54	5.37	0.19	2.42	3.63E-72
C3	887.374	141.778	2.637	9.40E-31
C3AR1	8.87	1.08	2.246	2.39E-56
C4orf3	86.36	17.27	2.257	4.24E-100
C5AR1	9.69	1.63	2.023	1.55E-55
C5orf15	47.199	8	2.421	3.58E-101
C5orf46	7.05	0.14	2.82	2.14E-64
C6orf1	35.631	7.81	2.056	2.60E-82
C6orf222	4.31	0.19	2.158	2.25E-33
C9orf89	39.151	6.14	2.491	1.55E-100
CA4	1.36	10.45	-2.278	4.67E-43
CA9	37.07	1.6	3.872	9.13E-45
CACNB3	17.881	1.72	2.795	3.03E-105
CALB2	6.41	0.19	2.638	1.46E-60
CALCRL	7.87	1.06	2.106	1.43E-66
CALD1	125.957	13.64	3.116	6.85E-74
CALHM2	20.41	3.23	2.34	5.02E-76
CALM2	557.141	79.291	2.797	2.36E-101

CALML4	34.489	5.13	2.533	4.20E-80
CALR	583.989	120.241	2.271	9.94E-89
CALU	78.351	15.42	2.273	3.28E-82
CAMK2D	16.59	3.36	2.012	5.76E-83
CAMK2N1	72.972	2.88	4.253	2.27E-137
CAMK2N2	1.15	11.41	-2.529	6.27E-61
CANT1	40.36	7.73	2.244	1.17E-95
CAP1	223.386	25.08	3.105	2.73E-105
CAPG	367.092	15.19	4.507	9.30E-106
CAPN2	152.81	28.21	2.397	8.34E-96
CAPN5	36.79	2.26	3.535	2.45E-95
CAPN8	71.169	1.22	5.023	8.72E-78
CAPN9	4.91	0.03	2.521	2.58E-51
CAPNS1	737.448	123.716	2.566	1.80E-96
CAPZA2	69.41	13.69	2.261	1.55E-83
CAPZB	302.564	36.921	3.001	9.97E-110
CARD11	20.12	0.47	3.845	4.51E-113
CARD16	28.34	1.54	3.53	9.59E-111
CARD6	7.45	0.99	2.086	2.98E-47
CARHSP1	91.81	19.77	2.16	4.69E-89
CASC4	48.509	9.02	2.305	2.29E-92
CASK	20.579	4.32	2.02	1.83E-93
CASP1	26.679	2.65	2.923	4.12E-83
CASP3	18.93	3.6	2.115	6.50E-104
CAST	112.228	21.35	2.341	1.61E-84
CAV1	42.261	5.06	2.836	1.71E-72
CAV2	51.282	9.6	2.302	3.11E-57
CBFB	29.13	5.96	2.114	1.08E-96
CBR1	57.531	13.1	2.054	3.09E-88
CBR3	6.37	0.52	2.278	3.65E-108
CBS	0.76	6.14	-2.02	6.38E-32
CCDC109B	12.3	0.86	2.838	9.89E-115
CCDC23	24.13	4.58	2.171	9.96E-106
CCDC3	9.46	1.16	2.276	1.58E-71
CCDC51	11.09	1.94	2.04	8.56E-125
CCDC71L	14.65	2.88	2.012	1.20E-81
CCDC74A	11.15	1.19	2.472	8.45E-95
CCDC80	108.638	8.91	3.468	6.59E-61
CCDC88B	26.21	4.42	2.328	2.69E-73
CCL13	3.78	0.17	2.031	9.48E-51
CCL15-CCL1	8.49	0.24	2.936	1.34E-69
CCL17	6.5	0	2.907	4.38E-55
CCL18	29.331	0.05	4.852	2.83E-88
CCL19	26.629	0.44	4.262	2.16E-50
CCL20	19.02	0.3	3.945	2.97E-76
CCL21	38.751	3.36	3.189	3.69E-38
CCL24	4.49	0	2.457	7.32E-55

CCL3	6.61	0.3	2.549	4.85E-68
CCL4	7.51	0.72	2.307	9.29E-61
CCL4L2	5.3	0.17	2.429	2.91E-61
CCL5	22.88	1.1	3.507	1.11E-87
CCNA2	5.14	0.36	2.175	1.67E-96
CCNB1	19.68	1.54	3.025	2.50E-119
CCNB2	8.88	0.23	3.006	1.08E-108
CCND1	56.68	10.99	2.266	5.13E-83
CCND3	64.35	11.98	2.332	1.87E-87
CCRL2	6.31	0.48	2.304	1.22E-88
CD109	4.49	0.15	2.255	2.56E-83
CD14	56.398	5.93	3.05	2.44E-80
CD163L1	4.25	0.26	2.059	2.01E-70
CD2	7.05	0.42	2.503	2.49E-67
CD248	42.789	3.72	3.214	4.64E-75
CD27	5	0.28	2.229	3.29E-57
CD276	82.452	11.4	2.751	1.36E-100
CD300A	11.79	0.69	2.92	6.72E-86
CD300LF	3.79	0.16	2.046	2.51E-67
CD37	26.42	2.76	2.866	6.35E-59
CD3D	9.89	0.41	2.949	6.08E-74
CD3E	11.78	0.71	2.902	3.91E-67
CD4	23.11	2.05	2.983	6.81E-78
CD47	90.917	16.02	2.433	3.71E-88
CD48	12.25	0.57	3.077	8.68E-67
CD52	76.968	1.88	4.759	5.37E-89
CD53	38.39	3.09	3.268	2.86E-66
CD55	103.042	5.82	3.931	6.80E-109
CD58	23.92	2.4	2.874	4.06E-99
CD59	442.826	72.67	2.591	6.61E-94
CD63	1573.868	349.051	2.17	1.78E-80
CD68	144.958	4.89	4.631	3.57E-102
CD7	5.64	0.41	2.236	2.61E-70
CD74	2261.712	99.477	4.493	4.13E-98
CD79A	6.81	0.2	2.702	6.09E-47
CD82	97.51	10.61	3.085	9.68E-111
CD83	6.43	0.63	2.188	3.04E-73
CD86	6.24	0.28	2.5	4.21E-76
CD9	252.807	33.62	2.874	1.10E-85
CD93	16.99	2.58	2.329	5.04E-72
CDA	18.71	0.38	3.836	7.52E-91
CDC20	15.07	0.22	3.719	1.04E-113
CDC42	155.05	27.87	2.434	4.77E-98
CDC42EP2	16.5	1.87	2.608	3.84E-103
CDC42EP3	18.01	2.61	2.397	3.17E-88
CDC42EP5	75.583	5.23	3.62	2.46E-95
CDCA3	6.93	0.69	2.23	6.05E-94

CDCA5	5.98	0.23	2.505	1.54E-110
CDCA7	8.01	0.62	2.476	5.91E-61
CDCA8	5.46	0.29	2.324	8.97E-104
CDCP1	22.02	2.65	2.657	5.72E-92
CDH11	35.309	0.89	4.264	4.86E-95
CDH13	3.82	0.19	2.018	1.04E-83
CDH17	6.6	0.08	2.815	4.59E-50
CDH3	50.882	0.42	5.191	1.15E-109
CDHR2	13.65	0.1	3.735	2.60E-83
CDHR5	9.46	0.65	2.664	1.68E-62
CDK1	12.16	0.33	3.307	4.62E-122
CDK2AP1	119.543	18.03	2.663	1.34E-110
CDK5	15.66	2.95	2.076	1.50E-113
CDKN2A	6.73	0.5	2.365	2.69E-45
CDKN2B	11.16	0.53	2.991	5.31E-79
CDKN2D	13.7	2.05	2.269	1.41E-101
CDKN3	10.02	0.33	3.051	1.89E-123
CDR2L	19.129	1.36	3.092	3.26E-110
CDS1	10.13	1.55	2.126	1.74E-94
CDT1	3.97	0.22	2.026	5.43E-90
CEACAM1	25.41	0.89	3.805	6.73E-115
CEACAM5	134.279	0.07	6.982	1.64E-84
CEACAM6	518.14	1.59	7.647	7.91E-111
CEACAM7	10.76	0.56	2.914	1.20E-43
CEBPB	58.969	12.16	2.188	8.01E-65
CECR1	17.579	2.96	2.23	1.36E-51
CEL	19.291	12250.049	-9.238	2.78E-70
CELA2A	4.53	10247.61	-10.856	2.71E-61
CELA2B	2	1188.23	-8.631	4.63E-57
CELA3A	47.431	32225.161	-9.378	1.01E-60
CELA3B	8.97	13424.678	-10.395	1.29E-70
CELP	0.18	187.415	-7.319	3.40E-94
CELSR1	10.59	0.49	2.96	1.74E-101
CEMIP	9.75	0.33	3.015	1.72E-84
CENPF	10.69	0.13	3.371	1.55E-89
CENPK	4.08	0.26	2.011	2.05E-94
CENPM	8.82	0.26	2.962	6.97E-91
CENPU	6.73	0.67	2.211	3.02E-93
CENPW	12.83	0.73	2.999	1.98E-117
CEP170	14.44	1.88	2.423	6.20E-90
CEP55	5.54	0.06	2.625	1.35E-107
CERCAM	53.94	9.42	2.398	2.07E-68
CERS6	12.12	1.56	2.358	9.26E-100
CERS6-AS1	22.389	2.91	2.581	1.73E-83
CES1	28.929	3.97	2.59	1.42E-47
CETN2	45.781	8.5	2.3	1.36E-111
CFB	130.598	21.97	2.518	1.40E-39

CFH	51.349	3.49	3.543	4.90E-88
CFI	58.169	13.36	2.043	2.56E-42
CFL1	1120.633	94.398	3.555	6.50E-117
CGB5	3.74	0	2.245	2.44E-34
CGREF1	7.55	0.71	2.322	2.21E-85
CH17-360D5	5.41	0.03	2.638	6.13E-77
CHAD	0.6	8.93	-2.634	3.89E-85
CHIC2	19.95	3.67	2.165	2.78E-110
CHIT1	5.46	0.13	2.515	1.45E-42
CHMP1B	32.61	6.37	2.189	3.25E-101
CHMP4B	174.03	30.399	2.479	8.98E-110
CHMP5	62.469	12.85	2.196	2.64E-100
CHN1	10.87	1.42	2.294	4.62E-81
CHST11	11.08	1.24	2.431	6.43E-69
CHST14	17.12	3	2.18	4.74E-102
CHST4	10.51	1.74	2.071	1.22E-36
CHSY1	15.54	2.05	2.439	2.07E-90
CIDEC	5.06	0.49	2.024	1.76E-27
CIITA	7.1	0.88	2.107	1.63E-54
CKAP2	5.71	0.59	2.077	3.72E-109
CKLF	91.113	7.03	3.52	6.53E-129
CKMT1A	19.99	1.7	2.959	1.51E-87
CKMT1B	20.06	1.58	3.029	2.30E-90
CKS1B	71.848	8.32	2.966	1.62E-112
CKS1BP3	5.69	0	2.742	9.88E-95
CKS2	38.569	1.47	4.002	2.43E-134
CLCF1	11.9	2.19	2.016	6.73E-56
CLDN11	7.1	0.48	2.452	2.38E-69
CLDN18	60.429	0.12	5.777	1.06E-62
CLDN2	39.829	2.29	3.633	3.04E-44
CLDN23	10.29	1.32	2.283	1.65E-62
CLDN4	279.75	41.671	2.718	1.48E-53
CLEC11A	26.099	2.47	2.965	2.16E-91
CLEC2B	10.32	0.97	2.523	2.87E-81
CLIC1	596.632	55.422	3.405	4.79E-113
CLIC3	24.421	0.3	4.289	4.33E-83
CLIC4	49.428	9.56	2.256	2.21E-64
CLIP3	9.21	1.23	2.195	2.25E-72
CLPS	20.97	38160.699	-10.762	3.75E-70
CLPSL1	0.11	44.6	-5.36	9.46E-38
CLRN3	8.07	0.05	3.111	6.96E-75
CLTB	208.976	26.29	2.944	2.38E-103
CLTC	115.824	23.94	2.228	1.17E-104
CMIP	39.069	5.83	2.553	9.31E-97
CMTM3	56.759	2.42	4.078	4.85E-106
CMTM6	43.17	6.12	2.633	8.22E-104
CMTM7	50.07	8.25	2.465	7.78E-74

CNIH1	81.007	18.15	2.098	6.33E-93
CNIH4	42.25	7.45	2.356	2.83E-102
CNN1	28.689	2.22	3.205	7.61E-64
CNN2	131.433	26.779	2.253	1.08E-58
CNP	49.48	11.34	2.032	1.64E-101
CNPY4	14.96	1.94	2.441	6.41E-101
CNTN1	5.76	0.57	2.106	8.85E-55
COA6	32.421	6.83	2.094	1.92E-115
COCH	1.47	9.69	-2.114	2.90E-40
COL10A1	78.569	0.07	6.217	1.93E-101
COL11A1	30.6	0.13	4.806	2.08E-77
COL12A1	52.189	1.94	4.177	3.98E-95
COL14A1	25.91	1.56	3.394	1.17E-72
COL15A1	30.879	5.37	2.323	6.65E-57
COL16A1	62.081	4.77	3.451	1.09E-90
COL17A1	62.141	0.08	5.869	2.46E-91
COL1A1	1201.648	12.61	6.465	2.17E-111
COL1A2	1166.361	18.25	5.922	5.25E-111
COL22A1	4.49	0.1	2.319	6.39E-66
COL3A1	1103.062	13.04	6.297	3.23E-110
COL4A1	129.624	9.91	3.582	2.54E-90
COL4A2	163.516	13.06	3.549	6.46E-95
COL5A1	115.936	2.59	5.026	2.06E-105
COL5A2	71.61	1.71	4.744	1.55E-108
COL6A1	179.955	35.779	2.299	2.74E-50
COL6A2	277.798	30.049	3.167	2.09E-70
COL6A3	188.118	12.97	3.759	3.89E-61
COL7A1	19.26	0.83	3.469	7.19E-74
COL8A1	25.989	0.34	4.332	1.50E-96
COL8A2	9.66	0.25	3.092	1.44E-86
COL9A2	7.89	0.9	2.226	1.54E-62
COLGALT1	50.509	11.31	2.065	1.31E-76
COMP	69.888	0.75	5.34	2.68E-93
COMT	144.867	33.481	2.081	7.82E-84
COPS8	39.871	8.03	2.178	2.97E-97
CORO1A	33.55	3.72	2.872	3.01E-65
CORO1C	38.66	5.31	2.652	1.51E-97
CORO2A	12.61	0.12	3.603	1.87E-120
COTL1	161.892	9.41	3.968	6.31E-119
COX6B1	346.785	80.37	2.096	7.35E-92
COX6B2	3.69	0.09	2.105	2.20E-49
COX7A1	52.138	10.15	2.253	2.72E-70
COX7A2	280.507	65.018	2.092	3.03E-96
COX7B	121.237	21.67	2.431	4.44E-115
CP	21.591	1.23	3.341	1.57E-36
CPA1	28.45	34590.781	-10.198	7.40E-64
CPA2	11.04	6821.625	-9.146	5.91E-66

CPA4	0.39	6.09	-2.351	1.76E-77
CPB1	40.419	15875.34	-8.582	4.26E-54
CPD	22.32	4.43	2.103	1.72E-90
CPNE2	23.769	5.06	2.031	3.38E-78
CPNE7	5.43	0.54	2.062	2.45E-67
CPPED1	8.69	1.14	2.179	1.28E-99
CPT1C	8.26	1.21	2.067	4.92E-64
CPVL	31.89	2.51	3.228	6.04E-79
CPXM1	17.19	0.76	3.37	3.88E-75
CPXM2	11.19	0.94	2.652	2.26E-74
CPZ	16.979	0.39	3.693	7.48E-110
CRABP2	50.369	0.87	4.78	7.16E-92
CRIM1	19.99	3.68	2.165	9.83E-79
CRIP1	528.074	5.46	6.356	1.40E-142
CRIP2	193.702	22.68	3.04	3.43E-85
CRISP3	15.401	0.35	3.603	2.61E-31
CRISPLD2	47.629	6.81	2.638	4.40E-62
CRNKL1	18.17	3.7	2.028	1.27E-118
CRP	35.429	3.59	2.989	1.33E-08
CRTAP	104.538	19.87	2.338	1.51E-80
CSDC2	1.79	15.56	-2.569	6.13E-52
CSF1R	18.1	2.26	2.551	8.93E-56
CSF2RA	8.73	0.47	2.727	1.07E-77
CSK	46.381	8.11	2.379	2.22E-92
CSNK1A1	86.677	18.17	2.193	6.54E-94
CSRP1	184.745	43.679	2.056	3.66E-74
CST1	100.092	0	6.66	1.18E-98
CST2	22.15	0.05	4.463	2.12E-84
CST6	12.58	0.2	3.5	1.28E-61
CST7	8.68	0.52	2.671	9.73E-77
CSTA	15.5	0.99	3.052	6.40E-69
CSTB	122.479	12.59	3.184	3.07E-107
CTB-191K22	39.191	1.57	3.967	3.66E-97
CTB-193M12	6.38	0.45	2.348	3.56E-113
CTB-31020	7.17	0	3.03	9.21E-56
CTB-50L17	0.18	6.56	-2.68	8.93E-125
CTC-425F1	9.59	1.01	2.397	2.60E-32
CTD-2008P7	0.23	9.98	-3.158	4.27E-127
CTD-2227E1	1.4	15.41	-2.773	1.81E-84
CTD-2231E1	36.78	7.56	2.142	3.00E-113
CTD-2377D2	7.4	0	3.07	7.10E-48
CTD-3252C9	18.539	2.64	2.424	1.26E-54
CTDSPL	24	4.74	2.123	2.14E-96
CTGF	172.804	25.029	2.739	1.57E-50
CTHRC1	109.303	0.89	5.867	2.75E-117
CTNNA1	196.678	40.63	2.247	3.08E-86
CTNNB1	155.319	29.84	2.342	1.62E-93

CTNNBL1	49.09	8.77	2.358	5.45E-120
CTRB1	63.941	28700.733	-8.788	1.94E-57
CTRB2	92.027	29919.531	-8.329	3.62E-55
CTRC	18.83	8000.641	-8.657	5.62E-59
CTRL	1.63	931.943	-8.471	1.80E-72
CTSA	166.883	30.92	2.395	5.65E-95
CTSB	658.664	58.012	3.483	1.10E-105
CTSC	149.935	14.76	3.26	3.20E-103
CTSD	819.442	129.238	2.655	6.29E-87
CTSE	321.393	0.6	7.655	1.96E-105
CTSH	338.543	43.98	2.916	1.01E-75
CTSK	122.683	3.46	4.793	6.34E-101
CTSL	115.407	26.319	2.091	5.09E-66
CTSO	22.721	4.54	2.098	2.30E-76
CTSS	137.472	4.72	4.597	3.11E-104
CTSZ	280.468	32.34	3.078	1.15E-98
CTTNBP2NL	10.86	1.19	2.437	3.54E-102
CTXN1	5.5	0.61	2.013	3.33E-61
CUZD1	1.83	866.946	-8.261	1.05E-83
CX3CL1	24	3.83	2.372	6.46E-65
CXCL10	6.13	0.13	2.658	1.47E-57
CXCL13	3.45	0.07	2.056	1.62E-34
CXCL14	88.401	3.05	4.464	3.23E-73
CXCL16	93.538	6.72	3.614	1.79E-119
CXCL3	6.73	0.68	2.202	1.09E-44
CXCL5	25.43	0.07	4.626	2.47E-70
CXCL6	7.41	0.95	2.109	1.45E-23
CXCL8	23.72	1.02	3.613	2.27E-48
CXCL9	4.9	0.09	2.436	1.27E-54
CXCR4	60.652	2.27	4.237	1.18E-93
CXCR5	5.69	0.64	2.028	1.17E-69
CYB5B	68.171	10.51	2.587	7.02E-113
CYB5R2	11.3	1.56	2.264	1.44E-90
CYB5R3	178.501	31.999	2.444	4.28E-93
CYBA	378.696	38.529	3.264	1.13E-76
CYBB	12.17	1.13	2.628	2.72E-58
CYBRD1	44.901	4.56	3.045	2.14E-75
CYCS	60.812	11.45	2.312	1.95E-99
CYGB	24.62	4.31	2.27	5.70E-69
CYP1B1	11.77	0.84	2.795	3.90E-57
CYP27A1	27.14	3.07	2.79	2.10E-91
CYP2C18	7.6	0.04	3.048	5.05E-76
CYP2S1	23.4	0.18	4.37	1.67E-92
CYP4F12	8.44	0.69	2.482	4.22E-60
CYP4F3	3.59	0.1	2.061	2.38E-64
CYR61	93.909	21.28	2.091	8.14E-31
CYSTM1	240.834	29.38	2.993	4.08E-96

CYTIP	7.62	1.09	2.044	9.97E-57
DACT1	9.57	0.65	2.679	1.19E-78
DACT3	4.37	0.33	2.014	1.87E-74
DAPP1	7.2	0.12	2.872	9.85E-94
DBI	242.795	48.19	2.309	2.45E-95
DBN1	49.428	6.71	2.709	3.81E-93
DBNDD2	71.54	14.87	2.193	2.85E-91
DBNL	87.729	18.34	2.198	5.67E-100
DCBLD2	13.34	2.21	2.159	2.10E-62
DCK	10.59	1.82	2.039	2.21E-90
DCN	473.722	53.382	3.126	2.03E-60
DCTPP1	43.98	9.37	2.117	3.03E-98
DDA1	43.989	7.82	2.351	3.55E-108
DDAH2	81.599	8.69	3.092	3.85E-99
DDR2	8.17	0.95	2.233	5.78E-64
DDX60	14.85	1.34	2.76	7.93E-97
DEGS1	35.55	7.43	2.116	5.15E-97
DENND2D	30.71	6.84	2.016	5.27E-76
DEPTOR	14.14	2.17	2.256	3.40E-98
DFNA5	8.97	1.13	2.227	3.08E-73
DGAT2	6.23	0.71	2.08	5.45E-55
DGUOK	69.791	13.62	2.276	4.66E-110
DHCR24	53.63	9.16	2.427	1.28E-56
DHCR7	22.65	4.65	2.066	2.04E-86
DHFR	7.93	0.78	2.327	4.93E-118
DHRS9	15.18	0.1	3.879	3.16E-62
DIO2	4.73	0.18	2.28	8.90E-77
DKK1	9.98	0.11	3.306	2.93E-66
DKK3	72.902	4.71	3.694	1.06E-110
DLGAP5	3.32	0.04	2.054	1.17E-96
DLL4	7.71	0.91	2.189	4.47E-84
DMBT1	25.779	0.28	4.387	6.11E-57
DNAJA4	18.77	3.82	2.036	7.14E-44
DNAJB1	86.15	16.63	2.305	2.07E-65
DNAJC15	18.911	3.3	2.211	6.08E-103
DNASE1L1	25.21	5.03	2.12	8.14E-105
DNASE2	47.93	10.83	2.048	4.84E-107
DNTTIP1	49.47	6.04	2.842	2.74E-116
DOCK11	7.74	0.61	2.441	2.43E-93
DOK2	6	0.74	2.008	1.07E-59
DOK4	38.991	6.39	2.436	9.36E-93
DOK5	5.14	0.2	2.355	1.49E-80
DPCD	28.249	5.44	2.183	7.43E-109
DPCR1	28.769	0.03	4.853	7.28E-62
DPEP1	17.179	113.118	-2.65	5.42E-57
DPP10-AS1	0.18	5.47	-2.455	1.00E-79
DPP3	40.341	8.52	2.119	2.82E-95

DPY19L1	12.64	2.05	2.161	2.02E-98
DPYSL2	32.64	3.64	2.858	3.63E-112
DPYSL3	48.04	1.37	4.371	1.51E-115
DRAP1	114.697	25.539	2.124	3.56E-103
DSE	8.95	1.19	2.184	1.42E-64
DSG2	43.51	6.27	2.614	7.68E-82
DUOX2	32.751	0.12	4.913	2.21E-70
DUOXA2	25.989	0.04	4.698	5.82E-67
DUSP5	23.169	2.15	2.94	8.96E-77
DUSP6	49.401	4.85	3.107	1.90E-114
DUSP7	14.26	2.33	2.196	1.08E-95
DYNC1H1	156.464	36.481	2.071	9.31E-95
DYNLL1	377.098	70.81	2.397	2.34E-99
DYNLRB1	237.6	39.7	2.551	3.45E-113
DYNLT1	136.588	21.19	2.632	7.86E-108
DYNLT3	26.75	4.35	2.375	9.86E-103
DYRK2	12.63	2.04	2.165	6.30E-108
E2F1	4.29	0.28	2.047	7.29E-100
E2F3	5.36	0.53	2.055	5.73E-104
EBP	77.718	16.829	2.142	3.84E-87
ECM1	45.629	1.59	4.17	4.65E-106
ECM2	6.25	0.57	2.207	1.00E-69
ECT2	11.54	0.81	2.792	1.33E-111
EDIL3	15.15	0.42	3.508	2.01E-104
EDNRA	11.46	0.35	3.206	2.65E-96
EFCAB11	7.14	1	2.025	2.26E-114
EFEMP1	48.06	11.11	2.018	1.17E-37
EFEMP2	81.43	10.52	2.839	1.49E-77
EFNA3	7.29	0.54	2.428	6.77E-86
EFNA4	16.88	2.04	2.556	4.79E-87
EFNA5	7.52	0.23	2.792	5.81E-110
EFNB1	42.2	2.58	3.593	1.96E-109
EFNB2	23.271	1.32	3.387	7.52E-124
EGF	0.46	15.85	-3.529	1.24E-109
EGLN2	60.921	14.03	2.043	1.17E-91
EGLN3	26.93	1.65	3.398	2.16E-75
EGR2	7.82	0.21	2.866	2.82E-82
EHBP1L1	47.649	6.68	2.663	2.91E-85
EHD1	40.009	5.17	2.733	3.06E-113
EHD2	50.299	10.3	2.183	2.26E-67
EID1	100.468	21.32	2.185	6.64E-86
EIF4E	35.5	7.86	2.043	1.88E-85
EIF5AP4	4.97	0	2.578	1.50E-141
EIF6	194.698	30.049	2.656	2.70E-113
ELF3	472.541	93.993	2.318	3.06E-37
ELF4	13.14	1.03	2.8	1.66E-122
ELK3	23.54	2.12	2.976	1.14E-109

ELOVL5	33.95	4.76	2.601	2.03E-87
ELOVL6	8.78	0.61	2.603	5.68E-87
EMB	16.429	0.55	3.491	1.58E-122
EMILIN1	69.261	5.9	3.348	2.82E-73
EMILIN2	12.15	1.15	2.613	5.34E-97
EMP1	108.187	7.3	3.718	6.11E-92
EMP2	34.539	4.69	2.643	5.90E-100
EMP3	85.692	5.33	3.776	1.34E-104
ENC1	33.69	7.09	2.1	1.70E-50
ENDOD1	16.46	1.94	2.57	4.44E-107
ENO1	620.461	80.481	2.931	1.15E-93
ENO2	32.901	3.14	3.034	1.05E-82
ENPP2	23.07	4.02	2.261	8.93E-40
SEMA3F	12.64	2.33	2.034	6.09E-78
LAP3	58.61	10.65	2.355	5.73E-89
HS3ST1	7.17	0.15	2.829	1.33E-93
MAD1L1	22.6	3.28	2.463	2.84E-94
LASP1	97.592	21.17	2.153	1.07E-92
TMEM176A	155.653	10.48	3.77	1.39E-97
TFPI	40.12	5.5	2.661	2.06E-61
PLXND1	69.3	13.77	2.251	1.81E-75
HCCS	17.6	3.53	2.038	1.88E-114
PRSS22	46.491	10.43	2.055	9.31E-35
SKAP2	20.269	1.69	2.983	7.08E-122
FAM214B	18.301	2.48	2.472	1.19E-98
WDR54	16.58	3.14	2.086	9.64E-96
RHBDD2	97.261	20.21	2.212	4.86E-95
ITGA3	135.082	9.7	3.669	2.32E-101
TMEM132A	23.02	2.18	2.917	8.31E-92
TNFRSF12A	153.223	32.41	2.207	7.89E-50
RALA	31.831	4.56	2.562	5.89E-122
ETV1	8.06	0.55	2.547	1.32E-99
USH1C	53.459	2.4	4.002	4.05E-104
GTF2IRD1	22.139	4.21	2.151	1.21E-83
SCIN	6.83	0.32	2.568	5.62E-81
PROM1	34.871	5.98	2.362	1.01E-40
GAS7	8.54	1.15	2.15	8.68E-67
TEAD3	20.15	3.97	2.089	7.62E-73
PLEKHG6	13.22	2.28	2.116	7.22E-73
ETV7	6.68	0.4	2.456	9.53E-92
PRSS3	93.37	2570.083	-4.768	1.21E-69
FYN	32.491	5.44	2.379	8.04E-82
MRC2	50.011	2.77	3.758	1.70E-91
TSPAN9	22.5	4.55	2.082	6.74E-80
PLAUR	101.279	1.78	5.201	4.39E-124
TYROBP	156.161	9.38	3.92	6.41E-85
TMEM159	31.48	4.68	2.516	1.63E-92

KDM5D	2.89	16.11	-2.137	0.00221
PSMC4	74.951	15.67	2.188	2.95E-104
MVP	198.513	44.64	2.128	1.89E-62
GPRC5A	141.19	0.95	6.188	7.80E-110
TACC3	18.29	1.61	2.886	1.21E-113
MTMR11	34.779	2.72	3.266	1.71E-83
WAS	8.85	1.01	2.293	1.38E-56
NPC1L1	8.03	0.19	2.924	4.01E-73
RALBP1	30.041	5.03	2.364	1.67E-113
WWTR1	29.1	6.18	2.068	9.00E-50
VSIG2	78.091	0.48	5.74	5.80E-100
MARCO	11.05	0.23	3.292	1.38E-53
SYT13	19.37	0.96	3.377	2.00E-92
RUNX3	4.34	0.26	2.083	9.18E-65
GRAMD1B	6.88	0.45	2.442	1.70E-82
PLEKHO1	38.22	2.73	3.394	4.15E-98
TYMP	75.118	7.94	3.09	2.92E-65
VIM	782.04	141.219	2.461	3.40E-62
FAS	11.36	1.95	2.067	2.04E-78
RNASET2	145.149	23.18	2.596	8.54E-97
SLAMF7	5.65	0.14	2.544	8.45E-76
TNFRSF1B	19.401	2.14	2.7	4.81E-88
GRN	359.039	33.369	3.389	5.56E-120
MAP2K3	48.331	10.58	2.091	3.42E-94
TMSB10	4461.768	253.298	4.133	1.03E-108
VCL	55.341	10.25	2.324	3.66E-86
TIMP2	228.269	27.319	3.017	1.03E-86
RPL26L1	38.21	8.64	2.024	2.50E-110
MAT2B	46.99	8.7	2.307	1.34E-98
VCAN	92.199	1.59	5.169	1.24E-102
MSR1	12.92	0.99	2.806	3.89E-69
RAI14	18.93	2.81	2.387	7.03E-86
PHF23	26.53	5.64	2.052	8.17E-107
PSMA4	148.476	31.78	2.189	2.27E-95
PARP3	17.99	2.64	2.383	5.95E-91
TNC	17.869	0.62	3.542	5.89E-76
LCP2	13.91	1.7	2.465	3.05E-60
KITLG	9.92	1.36	2.21	6.77E-67
VAMP3	65.14	11.7	2.381	6.83E-104
LTBP1	31.879	1.37	3.794	1.81E-102
RCN1	74.119	8.5	2.983	1.70E-117
HEXB	170.106	41.369	2.014	3.31E-86
LIMA1	65.58	11.88	2.37	2.68E-83
HOMER3	30.12	5.05	2.363	2.98E-73
RAD51	3.96	0.21	2.035	3.72E-101
THOC3	28.291	5.99	2.067	4.32E-98
MSMO1	44.289	7.19	2.467	2.16E-81

LAMA3	76.161	1.77	4.8	1.63E-121
ENTPD2	10.96	1.02	2.566	1.22E-84
LY75	12.52	0.52	3.153	2.84E-102
OPN3	13.11	1.43	2.538	8.37E-93
RELT	5.2	0.38	2.168	3.09E-86
PRDM1	6.07	0.4	2.336	1.76E-90
LAMC2	128.684	0.94	6.063	2.13E-118
TBXAS1	21.681	1.24	3.34	9.60E-103
MXD1	10.54	1.55	2.178	1.08E-74
SLC2A3	21.88	2.32	2.785	2.89E-63
STYK1	4.84	0.04	2.489	4.14E-97
GNA15	15.43	0.37	3.584	3.66E-116
MRPS35	38.239	8.15	2.1	2.65E-110
GPC1	38.419	8.38	2.071	1.33E-56
SPA17	9.53	1.55	2.046	2.57E-111
SLC12A2	17.39	2.11	2.564	1.35E-77
FAR2	6.23	0.5	2.269	3.27E-101
SLC9A3R2	99.421	18.34	2.376	1.08E-82
OAT	46.029	9.55	2.156	7.86E-81
MYLK	29.431	3.19	2.861	1.85E-59
TBC1D1	56.281	12.53	2.082	8.57E-85
TIE1	11.95	1.71	2.257	1.57E-66
SPI1	24.71	2.28	2.971	5.62E-71
PFKP	74.749	4.59	3.76	3.83E-116
TNFRSF1A	124.862	24.271	2.316	2.39E-81
PKM	754.04	48.539	3.93	4.92E-127
RHOA	358.318	73.541	2.269	1.61E-87
HYAL2	51.828	9.6	2.317	6.49E-94
IFI35	49.148	6.24	2.792	2.37E-104
PITX1	13.09	0.08	3.706	3.21E-86
VPS35	38.39	8.48	2.055	7.65E-98
MAOB	26.489	2.76	2.87	1.07E-83
PLA2G10	12.41	1.98	2.17	1.31E-43
SLC44A1	22.751	4.07	2.228	1.48E-92
FSTL3	62.37	13.13	2.165	1.91E-33
ST6GALNAC1	27.27	0.41	4.326	4.31E-92
OSBPL3	14.23	1.45	2.636	2.38E-88
MAP4K4	43.03	4.79	2.927	4.27E-110
WDR1	155.481	24.03	2.644	1.30E-104
LMCD1	58.932	7.95	2.743	5.11E-82
FCGR2B	12.41	0.57	3.094	3.15E-67
STK10	18.1	2.72	2.36	3.89E-98
NDE1	16.49	1.57	2.767	4.74E-111
MRVI1	8.81	1.09	2.231	1.65E-66
MCM2	8.27	1.18	2.088	5.31E-98
GSDMB	35.941	6.99	2.209	4.85E-44
PTGS2	4.81	0.1	2.401	4.23E-62

ST6GAL1	19.67	2.16	2.71	1.91E-76
NOTCH3	31.6	3.85	2.749	8.08E-79
MGLL	65.639	2.7	4.171	1.71E-119
ZNF532	16.13	2.82	2.165	1.25E-66
HACD3	36.661	7.33	2.177	7.10E-108
SRI	154.835	34.77	2.123	1.02E-82
SEMA3C	18.581	0.29	3.924	9.85E-86
FOSL2	41.08	8.22	2.19	6.06E-51
FSCN1	54.451	2.14	4.142	4.89E-113
RAB7A	148.024	31.851	2.182	1.21E-97
MCM6	12.09	1.67	2.294	1.02E-115
SPAG5	8.6	1.13	2.172	1.14E-95
MCAM	42.39	8.46	2.197	6.63E-57
UBE2T	12.37	0.31	3.351	4.16E-142
IL4R	45.73	9.91	2.099	3.75E-48
PAK3	1.07	7.76	-2.081	1.35E-58
GPR137B	14.97	1.2	2.86	1.47E-113
FBLN1	135.147	26.85	2.289	1.41E-39
FAP	35.809	1.58	3.835	2.74E-85
GNB1	155.47	29.459	2.361	5.80E-98
TP53INP2	13.53	2.52	2.045	3.63E-85
FKBP7	15.401	1.59	2.663	3.17E-103
LXN	36.44	4.4	2.794	2.31E-99
MKNK1	18.97	95.312	-2.27	4.05E-87
PAFAH1B3	35.559	6.89	2.212	7.88E-96
MOXD1	15.66	0.67	3.318	1.11E-83
STX7	20.649	4.23	2.049	4.37E-78
KEAP1	49.81	11.65	2.006	1.64E-107
PTPRH	21.23	1.92	2.928	2.27E-80
PSEN1	35.661	6.37	2.315	3.49E-112
NDC80	4.4	0.1	2.295	8.83E-110
PTPRC	10.19	1.03	2.463	2.09E-49
STK17B	10.36	1.62	2.116	1.80E-74
FYB	5.69	0.3	2.364	6.31E-65
NFE2L1	89.009	16.39	2.372	5.53E-100
GSK3B	14.97	2.97	2.008	1.93E-109
ITGB5	161.769	11.21	3.737	1.72E-114
PLOD1	69.69	15.34	2.113	1.72E-84
OXCT1	9.28	1.53	2.023	1.05E-86
FAT1	79.439	14.3	2.394	4.74E-77
GSTP1	765.627	134.084	2.505	2.85E-64
KIF3C	5.42	0.6	2.005	8.17E-83
SLC25A24	18.5	2.7	2.398	7.71E-89
IGSF9	6.13	0.78	2.002	3.74E-52
MGST2	100.037	21.681	2.155	3.52E-102
MAST2	18.439	3.73	2.039	5.28E-85
MRPL28	60.379	13.39	2.093	4.15E-95

ERO1LB	6.92	54.64	-2.813	1.28E-49
HSD17B2	45.439	7.47	2.455	1.30E-34
LAT2	11.44	1.5	2.315	4.86E-65
NOX4	8.15	0.2	2.931	1.35E-89
PPP1R15A	55.541	8.69	2.545	9.28E-78
HSD17B14	10.47	1.05	2.484	9.00E-73
FTL	4414.7	722.322	2.61	7.11E-74
PIGS	40.201	9.26	2.006	1.47E-90
MMP2	258.567	16.99	3.851	1.13E-72
LPCAT2	10.76	1.33	2.335	4.69E-88
PTHLH	5.1	0.21	2.334	1.05E-75
PHACTR3	5.52	0.14	2.516	7.11E-59
SULT2B1	6.93	0.22	2.7	1.21E-73
TPX2	9.55	0.09	3.275	1.88E-116
FER1L4	11.43	0.43	3.12	8.55E-59
EPB41L1	48.68	8.39	2.404	6.39E-85
SMOX	20.559	4.28	2.03	2.64E-78
FKBP1A	196.501	46.189	2.065	7.87E-84
SLC4A11	5.85	0.21	2.501	8.22E-87
F11	1.79	12.65	-2.291	7.18E-57
OAS1	45.781	1.4	4.285	8.72E-115
PXN	64.391	14.77	2.052	3.26E-83
FXYD5	309.521	9.04	4.951	1.81E-126
FXYD3	599.202	19.41	4.878	1.01E-105
HEPH	15.57	0.54	3.428	1.16E-104
SLC9A1	31.741	4.74	2.512	1.82E-98
RGS1	62.738	4.24	3.605	2.33E-62
NDUFB2	235.127	57.308	2.018	1.59E-88
ICAM1	35.37	2.91	3.218	4.73E-75
LYZ	837.357	12.59	5.947	5.63E-90
P3H2	9.2	1.31	2.143	3.47E-66
PLEKHG2	15.6	2.93	2.079	4.87E-64
NAT14	19.521	3.55	2.173	9.97E-77
SEL1L3	72.639	16.33	2.087	6.79E-61
NLRP1	18.81	3.69	2.079	1.30E-47
PSME1	259.663	50.091	2.351	5.73E-92
EZR	222.706	29.531	2.873	1.13E-95
MYL6	2224.244	394.258	2.493	8.27E-80
UNC13D	24.57	4.13	2.317	1.32E-52
TGFB2	7.06	0.95	2.047	4.88E-51
GABRP	26	0.12	4.591	2.01E-70
EPB41L4B	9.07	48.67	-2.302	1.52E-60
PTGS1	6.72	0.66	2.217	1.99E-76
TBC1D2	12.84	1.06	2.748	1.08E-113
SMIM24	50.021	1.58	4.306	2.56E-80
TREM2	23.801	0.97	3.654	1.60E-82
ITPR3	30.29	4.61	2.48	1.15E-68

SCD	36.279	2.29	3.502	2.60E-86
PRTFDC1	8.49	1.09	2.183	6.10E-103
TSPAN15	88.462	7	3.483	1.34E-96
H2AFY2	14.54	2.69	2.074	2.65E-81
MISP	49.169	0.21	5.374	2.24E-151
MMP11	266.242	0.56	7.42	2.96E-119
SUSD2	9.43	1.41	2.114	4.25E-54
GGT5	71.649	8.52	2.932	5.38E-83
SEC14L2	20.639	1.54	3.091	1.73E-111
SNRPD3	107.098	20.831	2.308	1.98E-113
MFNG	11.18	1.74	2.152	7.88E-76
SH3BP1	12.3	1.51	2.406	7.17E-91
LGALS1	962.604	51.76	4.191	2.40E-97
PIK3IP1	34.659	3.85	2.878	3.42E-94
SLC5A1	11.07	0.43	3.077	7.76E-78
KDELR3	32.43	5.23	2.424	5.27E-86
TIMP3	151.208	27.67	2.408	1.81E-52
TOM1	46.301	9.88	2.12	8.24E-93
HMOX1	21.31	1.77	3.01	2.47E-80
TSPO	295.988	34.811	3.052	1.05E-78
PDGFB	14.09	2.39	2.154	1.22E-76
KIAA0930	41.469	7.55	2.312	8.46E-91
NCF4	10.46	1.28	2.329	4.07E-61
KCTD17	16.53	1.89	2.601	8.33E-104
RBX1	51.771	10.26	2.229	1.13E-109
PYGL	16.829	2.82	2.223	3.05E-68
PLEK2	51.588	2.68	3.837	3.35E-88
SPTLC2	15.27	2.16	2.364	4.26E-124
RIN3	12.14	1.12	2.632	6.54E-98
LGMN	120.358	14.69	2.951	1.26E-109
ERH	112.352	23.61	2.203	5.22E-104
HIF1A	65.462	9	2.733	3.51E-83
GSKIP	19.51	3.42	2.214	1.69E-105
PSME2	232.985	49.752	2.205	1.77E-85
SEC23A	19.231	3.09	2.306	2.64E-88
GSS	35.031	5.68	2.431	1.58E-122
MMP9	21.091	0.11	4.315	1.69E-95
PYGB	80.12	10.15	2.863	2.49E-105
PROCR	26.259	3.1	2.733	5.21E-92
SLMO2	36.811	7.58	2.14	2.67E-98
SLCO4A1	14.9	2.39	2.23	3.37E-47
SLC17A9	14.67	2.15	2.315	1.21E-49
PTK6	18.84	0.94	3.354	1.75E-75
RASSF2	7.08	0.77	2.191	1.04E-62
SLC52A3	6.58	0.45	2.386	1.30E-89
FERMT1	15.519	0.69	3.289	5.84E-106
MYL9	325.564	26.55	3.567	5.80E-88

HCK	13.07	0.93	2.866	4.49E-72
MAPRE1	56.308	12.29	2.108	5.16E-96
WFDC2	202.348	7.9	4.514	5.11E-84
FAM83D	4.23	0.1	2.249	4.07E-77
SYNDIG1	3.69	0.1	2.092	4.85E-79
MYL12A	568.414	89.771	2.649	1.42E-81
SMAD7	16.88	3.46	2.003	6.61E-70
LIPG	6.07	0.45	2.286	7.40E-78
MXRA5	35.26	1.81	3.69	9.43E-75
VSIG1	11.39	0.03	3.588	2.64E-57
STS	6.38	0.8	2.036	9.82E-88
PGRMC1	74.042	13.96	2.327	5.26E-106
PLS3	72.519	9.1	2.864	8.81E-101
RENBP	11.17	0.93	2.657	1.50E-83
PIM2	12.63	1.79	2.288	1.60E-89
PGK1	287.954	44.319	2.673	2.78E-98
SMS	63.65	8.84	2.716	9.50E-126
RP2	6.56	0.85	2.031	7.33E-105
VGLL1	4.5	0.22	2.173	3.17E-47
TIMP1	1414.424	46.951	4.884	6.89E-107
MAGED2	117.221	26.099	2.125	3.03E-88
SRPX2	16.22	1.27	2.923	8.83E-81
GLA	23.48	4.83	2.07	2.79E-100
KLF5	67.302	3.2	4.023	1.20E-97
STK24	85.07	18.43	2.147	3.04E-97
PARP4	30.549	5.95	2.183	3.17E-82
TSC22D1	110.438	13.69	2.923	4.99E-85
OLFM4	322.241	29.67	3.398	5.34E-12
MSLN	189.755	0.12	7.412	1.22E-109
MAPK3	98.659	13.88	2.744	2.74E-116
GDPD3	7.89	1.11	2.075	8.43E-61
N4BP1	19.18	4.04	2.001	4.96E-93
PLA2G15	15.34	3.04	2.016	1.31E-91
FA2H	24.289	2.36	2.912	1.05E-73
HCFC1R1	122.827	17.37	2.753	2.68E-99
SLC7A5	15.76	1.38	2.816	7.03E-64
PIEZO1	101.997	13.12	2.867	1.26E-75
TCEB2	401.79	73.099	2.442	1.30E-104
TOX3	7.52	0.91	2.157	2.10E-65
PYCARD	58.822	4.28	3.502	3.49E-112
TMC5	58.032	0.87	4.98	1.47E-100
RAB11A	195.117	30.46	2.64	3.79E-109
VPS18	12.84	2.26	2.086	2.45E-117
LAPTM4B	59.56	11.69	2.255	5.38E-88
PLAT	123.237	3.06	4.935	2.37E-110
WISP1	8.46	0.17	3.015	5.55E-79
NDRG1	132.807	30.311	2.095	1.20E-63

STMN2	11.05	0.78	2.759	3.61E-63
SQLE	28.019	3.22	2.782	1.82E-91
TNFRSF10A	8.71	0.87	2.376	6.05E-87
FGL1	1.97	132.697	-5.492	2.48E-67
MAN2B1	81.199	16.731	2.213	2.31E-91
KCNN4	78.531	0.39	5.838	1.05E-117
NUCB1	255.52	59.531	2.083	1.70E-80
RELB	25.64	5.14	2.117	1.21E-57
PPP1R13L	62.111	13.3	2.142	1.02E-53
RNASEH2A	18.04	3.75	2.003	2.28E-105
IL4I1	4.7	0.24	2.201	2.47E-74
PPP6R1	41.879	8.07	2.241	1.55E-105
GPI	178.02	34.379	2.339	7.79E-96
TBCB	122.904	21.58	2.456	9.37E-102
SLC1A5	62.782	10.51	2.47	3.21E-75
TJP3	39.129	4.83	2.783	6.21E-62
TGFB1	50.001	5.3	3.017	3.00E-89
PLIN3	58.351	6.89	2.911	1.05E-108
MYH14	79.489	7.66	3.216	1.64E-96
NKG7	7.93	0.58	2.499	7.16E-70
ETFB	126.263	27.24	2.172	1.30E-95
MEIS3	12.9	1.62	2.407	5.26E-70
FAM83E	30.95	0.5	4.413	4.34E-108
PLEKHA4	27.62	4.79	2.305	9.22E-78
PPP2R1A	177.637	37.579	2.211	2.92E-98
TFPT	24.989	3.36	2.576	1.91E-115
PDE4C	16.959	0.93	3.218	5.69E-60
LSR	212.437	39.371	2.402	1.86E-71
HPN	8.77	103.278	-3.416	1.39E-69
ETHE1	67.199	14.77	2.113	5.57E-79
SMG9	40.481	7.11	2.355	9.33E-101
PON2	66.611	15.44	2.04	2.40E-74
MTPN	52.45	7.12	2.719	3.37E-114
H2AFV	110.43	26.37	2.025	5.03E-101
MET	23.73	2.31	2.901	4.77E-82
WNT2	4.96	0.02	2.547	1.45E-78
VIPR2	0.45	5.9	-2.251	6.43E-93
STX1A	28.561	5.24	2.244	2.42E-57
HSPB1	950.537	189.243	2.322	1.07E-40
NUDT1	29.3	3.52	2.745	4.87E-123
PCOLCE	96.228	16.89	2.442	7.22E-62
SERPINE1	89.052	6.66	3.555	1.44E-68
RPA3	28.869	5.61	2.176	6.07E-110
SFRP4	80.147	0.97	5.364	2.40E-93
GIMAP2	11.42	0.75	2.827	2.04E-93
TMEM176B	227.685	15.88	3.76	8.84E-94
LIMK1	24.319	4.17	2.292	1.38E-97

SLC1A1	11.05	0.79	2.751	6.94E-76
TRIM14	13.81	0.86	2.993	8.11E-120
TGFBR1	17.91	2.49	2.438	9.30E-87
PTGR1	40.411	7.81	2.233	1.23E-79
PDLIM1	187.571	36.549	2.328	8.42E-71
UNC5B	15.14	1.35	2.78	7.46E-102
SPOCK2	10.46	0.59	2.849	5.54E-78
MICU1	36.821	6.65	2.306	6.16E-122
LIPA	43.8	7.31	2.431	1.28E-83
LHPP	16.97	3.45	2.014	9.48E-102
SH3PXD2A	16.87	2.88	2.203	1.93E-81
UBE2S	30.279	2.36	3.219	9.77E-134
TSPAN14	46.291	10.7	2.015	1.98E-77
KRT23	15.35	0.63	3.326	9.53E-57
RAPGEFL1	19.721	3.45	2.219	1.43E-54
P2RX1	1.63	24.989	-3.305	1.08E-103
HOXB6	10.88	0.44	3.044	6.35E-90
PFN1	571.733	87.638	2.692	2.27E-96
ICAM2	28.829	5.71	2.152	1.03E-84
LGALS3BP	434.345	26.359	3.992	7.01E-132
RAB5C	120.977	26.07	2.172	9.83E-96
VAT1	106.661	25.389	2.028	2.76E-77
PPY	64.771	13.56	2.175	1.31E-11
FAM20A	9.83	1.03	2.415	1.54E-63
MMD	9.86	0.96	2.47	8.02E-94
PMP22	68.702	8.27	2.911	1.51E-80
RAB34	58.712	3.93	3.598	7.13E-99
TMEM33	20.45	3.74	2.178	2.26E-104
ODAM	4.71	0.17	2.287	5.55E-54
NMU	10.41	0.03	3.47	4.61E-79
KIAA1211	4.38	0.13	2.251	6.72E-80
INPP4B	12.29	0.75	2.925	1.37E-96
KLHL5	14.51	2.52	2.14	1.49E-71
HTATIP2	63.592	10.19	2.529	4.57E-99
MTCH2	58.85	12.2	2.181	2.40E-104
UNC93B1	53.319	7.68	2.646	7.24E-94
MS4A6A	56.68	5.53	3.143	7.33E-65
MS4A4A	13.74	1.81	2.391	1.49E-52
ST3GAL4	28.31	4.22	2.489	7.19E-63
FOLR1	11.8	1.57	2.316	2.10E-42
PANX1	10.72	1.9	2.015	7.82E-95
IL10RA	8.65	1.27	2.088	3.50E-54
SLC15A3	70.141	3.35	4.032	3.58E-109
MDK	367.397	10.05	5.059	4.01E-131
SLC22A18	40.26	5.88	2.584	2.31E-44
P3H3	26.891	5.7	2.058	1.09E-71
SELPLG	26.299	0.69	4.014	2.07E-107

KCTD10	21.54	4.36	2.072	2.16E-90
GLI1	4.3	0.32	2.005	4.89E-69
FOXM1	8.32	0.55	2.588	1.17E-92
OAS3	12.65	0.89	2.852	4.82E-94
OAS2	16.549	0.48	3.568	2.20E-83
MGP	633.762	71.288	3.134	7.64E-62
RASAL1	13.56	1.01	2.857	5.11E-78
VDR	13.46	0.75	3.047	6.10E-119
GAPDH	2426.564	232.404	3.379	8.80E-106
TPI1	461.791	92.661	2.305	4.52E-84
PTPN6	47.698	10.23	2.116	1.47E-80
NEDD9	21.5	3.13	2.446	6.24E-87
NCOA7	28.34	4.06	2.536	9.36E-79
STK38	25.801	4.47	2.293	2.60E-96
MCM3	30.99	6.32	2.128	3.48E-104
RAB23	6.89	0.63	2.275	1.29E-80
VNN1	9.43	0.34	2.96	1.71E-60
VNN2	7.15	0.51	2.432	2.10E-55
PERP	88.603	10.81	2.924	9.73E-103
MDFI	27.57	0.88	3.926	4.59E-110
TFEB	23.07	4.54	2.119	1.53E-84
PTK7	55.008	6.19	2.962	3.61E-87
LAMA4	25.88	1.42	3.473	4.20E-112
LY86	9.98	0.5	2.872	1.07E-73
KIF20A	4.05	0.07	2.239	3.20E-103
PFDN1	58.809	12.02	2.2	7.81E-113
HBEGF	16.88	1.17	3.043	6.73E-92
LOX	14.06	1.18	2.788	1.09E-66
SPARC	2167.775	49.49	5.425	8.01E-114
THBS4	38.869	6.37	2.436	1.03E-42
LMNB1	13.37	1.23	2.688	5.32E-114
SUB1	175.374	37.83	2.183	6.43E-98
GNPDA1	24.01	4.33	2.23	1.08E-111
H2AFY	142.734	20.22	2.76	1.09E-118
PDGFRB	63.689	8.34	2.792	3.45E-73
SERPINI2	0.51	192.497	-7.002	3.01E-87
GNAI2	214.182	24.07	3.102	2.92E-106
GNB4	6.48	0.68	2.155	2.28E-75
HHLA2	6.82	0.36	2.524	8.47E-58
MOB1A	34.671	5.55	2.445	6.00E-103
ITGB6	50.251	6.59	2.755	2.50E-49
PSMD14	35.461	6.32	2.316	1.54E-115
PDE1A	5.57	0.62	2.02	8.33E-73
RTN4	169.494	34.09	2.281	2.60E-90
REG1A	555.098	34447.221	-5.953	7.32E-48
FN1	978.955	17.23	5.748	1.81E-102
STAT1	53.578	8.2	2.569	7.65E-84

IGFBP5	189.755	8.7	4.298	1.34E-83
TXNDC9	20.391	4.24	2.029	9.24E-107
PDCL3	20.609	3.99	2.115	6.22E-117
IL1R2	12.19	0.8	2.873	2.18E-62
FHL2	116.331	3.32	4.763	1.59E-131
MLPH	121.439	11.37	3.307	1.71E-82
UXS1	26.88	5.91	2.012	1.57E-97
PROC	4.39	0.23	2.132	5.35E-77
PLEKHB2	39.229	7.52	2.239	1.47E-106
QPCT	21.791	1.57	3.149	4.05E-76
SDC1	144.567	5.41	4.505	6.92E-113
KYNU	11.71	0.22	3.381	1.35E-108
WIPF1	23.591	2.53	2.8	5.97E-80
PLEK	6.57	0.56	2.279	1.20E-54
RND3	24.13	1.89	3.12	2.06E-106
SUMO1	103.17	16.939	2.538	9.87E-121
EPHA4	5.42	0.42	2.177	2.03E-86
PRRX1	17.43	1.05	3.168	2.99E-75
SCP2	138.591	30.311	2.156	1.51E-90
LRRC42	25.08	4.52	2.24	1.25E-103
ICMT	31.41	6.61	2.09	1.10E-103
QSOX1	190.216	35.971	2.371	1.44E-81
RAP1A	50.86	11.27	2.08	1.25E-80
RNF19B	26.031	3.04	2.742	2.83E-111
NCF2	19.11	1.02	3.315	2.70E-70
GADD45A	34.539	6.36	2.272	1.65E-85
MAP7D1	70.609	8.95	2.847	4.73E-107
SIPA1L2	9.07	1.48	2.022	9.96E-70
MFAP2	74.222	9.34	2.863	1.69E-59
RGS4	6.96	0.74	2.194	1.16E-50
GBP3	25.37	1.89	3.19	1.37E-76
GBP1	16.09	1.72	2.652	4.32E-70
GALE	67.982	7.28	3.059	4.79E-87
P3H1	35.72	6.92	2.213	2.10E-82
SLC2A1	131.908	2.82	5.121	8.91E-117
PRDX1	363.295	44.711	2.994	9.35E-107
TSPAN1	258.459	2.14	6.369	3.88E-133
HSD11B1	8.27	0.57	2.562	5.17E-68
IRF6	29.96	6.6	2.026	9.35E-72
STMN1	104.162	8.49	3.47	3.27E-118
NEK2	4.93	0.05	2.498	5.50E-102
MUC5B	39.929	0.87	4.452	7.16E-51
NRP2	21.869	0.65	3.793	4.41E-115
RAB32	31.769	3.84	2.759	3.96E-88
MYL12B	545.108	78.08	2.788	1.93E-98
SPP1	540.218	62.439	3.093	2.35E-30
RARRES1	32.89	2.6	3.235	5.17E-55

PPL	27.41	3.86	2.547	4.23E-73
NDUFB3	78.88	15.899	2.241	7.06E-110
GDA	11.12	0.45	3.063	8.37E-85
ITGB1BP1	62.751	11.22	2.383	9.00E-105
PTBP3	25.05	3.96	2.393	2.68E-103
PPP2R4	113.873	23.93	2.204	3.92E-91
PHF19	17.59	2.29	2.498	4.11E-110
PGF	12.19	1.7	2.288	3.89E-78
IFI27L2	108.157	8.61	3.506	3.69E-118
NPC2	284.344	45.901	2.605	1.70E-97
LTBP2	28.15	2.49	3.062	1.39E-83
GPR68	5.54	0.23	2.411	4.37E-90
EPCAM	327.919	53.341	2.598	4.05E-76
IFIT3	17.019	1.81	2.681	1.34E-77
IFIT2	6.98	0.68	2.248	4.82E-82
GNA13	17.53	3.17	2.152	1.74E-93
HOXB5	5.12	0.28	2.257	6.11E-90
PLEKHG1	5.85	0.47	2.22	1.45E-95
PDZD11	43.661	8.47	2.238	2.47E-121
PLXDC2	16.381	0.87	3.216	1.56E-90
HSPH1	45.879	9.95	2.098	5.32E-75
TGFBI	168.791	7.64	4.297	4.27E-96
PLS1	30.149	2.64	3.097	6.11E-90
GLT8D2	14.05	1.02	2.897	3.94E-87
PDLIM2	56.96	7.61	2.751	1.76E-93
SCPEP1	47.679	9.06	2.275	1.44E-75
NCAPH	4.01	0.1	2.187	2.00E-104
PLBD1	54.518	7.64	2.684	2.60E-67
NAA50	52.008	12.11	2.016	1.28E-84
FABP3	13.16	0.87	2.921	7.99E-81
TNFSF10	51.31	8.1	2.523	3.25E-81
TMEM54	100.663	12.2	2.945	2.48E-102
SASH3	7.28	0.76	2.234	1.55E-51
FMOD	39.799	5.06	2.751	1.88E-84
7-Sep	87.147	13.92	2.563	3.47E-108
INHBA	15.58	0.09	3.927	4.36E-99
FKBP9	74.63	17.6	2.024	2.86E-89
TWIST1	5	0.2	2.322	2.42E-68
GLIPR2	20.831	1.44	3.161	4.01E-85
SPINK4	3.54	0.1	2.045	4.21E-27
PLAU	86.222	1.97	4.876	1.36E-103
SRGN	94.208	7.89	3.421	1.72E-79
P4HA1	29.321	6	2.115	2.01E-68
ZWINT	14.47	1.25	2.781	8.12E-116
HVCN1	6.69	0.57	2.292	6.44E-77
SSPN	10.79	1.1	2.489	5.74E-86
GIPC1	137.653	17.33	2.919	1.16E-105

OPTN	60.938	13.56	2.089	3.79E-91
NCKAP1L	6.88	0.69	2.221	1.82E-56
MMP19	12.4	2.09	2.117	1.97E-46
ORMDL2	58.318	12.1	2.179	3.07E-103
LRP1	90.452	11.01	2.929	1.15E-89
TUBA1B	538.274	60.681	3.128	1.34E-99
HJURP	3.54	0.08	2.072	3.52E-94
NMI	23.581	3.06	2.598	4.07E-105
TNFAIP6	7.93	0.09	3.034	1.23E-86
G0S2	25.751	5	2.157	3.93E-31
PI3	41.409	0.33	4.995	2.25E-88
SLPI	414.146	9.37	5.323	4.80E-96
WFDC3	5.9	0.26	2.453	5.34E-61
SDC4	194.819	26.87	2.813	1.96E-74
ZNFX1	17.12	2.44	2.397	2.50E-113
PTGIS	11.76	0.81	2.818	2.93E-61
SNAI1	7.83	0.94	2.186	3.30E-66
PMEPA1	117.099	9.77	3.455	2.48E-81
RBPJL	0.14	374.597	-8.364	5.49E-130
PEPD	47.179	10.63	2.051	1.45E-92
PAIP2B	1.31	13.6	-2.66	9.53E-93
POF1B	11.8	1.57	2.316	1.32E-65
LYPD3	5.22	0.46	2.091	8.48E-60
F13A1	14.32	2.34	2.198	8.90E-46
SPDEF	14.83	0.14	3.796	1.49E-86
GNMT	0.7	176.9	-6.709	1.15E-95
TREM1	11.81	0.21	3.404	6.22E-80
SOX4	21.19	3.3	2.368	2.27E-76
RUNX2	5.25	0.22	2.357	3.10E-89
LRRFIP1	61.252	10.92	2.385	5.75E-91
MT1G	60.748	259.717	-2.078	1.85E-17
IRF1	41.821	5.33	2.758	2.57E-79
6-Sep	18.6	1.82	2.797	8.30E-93
PTGER2	4.82	0.24	2.231	4.31E-83
PPDPF	489.952	93.299	2.38	1.40E-74
FOSB	11.3	1.27	2.438	5.87E-28
SNRPD2	293.333	70.521	2.041	6.52E-86
RTN2	13.45	2.14	2.202	1.72E-100
VASP	128.898	9.57	3.619	6.65E-116
SDCBP2	76.558	6.02	3.466	3.68E-72
SNRPB	161.075	29.06	2.431	2.23E-105
LAMP5	15.87	0.09	3.952	5.37E-97
SNRPB2	44.09	8.97	2.177	5.34E-108
FAM110A	11.14	1.85	2.091	1.12E-86
ID1	104.437	14.88	2.731	5.54E-67
RALY	128.043	30.819	2.02	8.91E-92
ROMO1	213.234	49.511	2.085	2.01E-89

PSMB2	56.519	12.74	2.066	1.62E-106
RBM42	63.848	14.67	2.049	4.39E-96
KIRREL2	1.34	23.74	-3.402	1.54E-89
HCST	20.959	1.71	3.019	4.48E-83
PRDX5	342.414	80.66	2.072	1.58E-88
RRAS	114.126	11.31	3.225	2.10E-101
GLIS2	22.611	4.43	2.12	7.94E-56
IFI6	300.765	10.41	4.725	5.85E-99
EVI2A	8.8	0.43	2.777	1.05E-75
TRAF2	16.32	2.78	2.196	2.16E-104
TSPAN8	343.198	12.72	4.649	1.87E-82
KLF2	31.051	3.85	2.724	7.40E-69
UQCR11	169.494	41.489	2.005	3.20E-93
PKMYT1	10.88	0.69	2.813	3.95E-97
TICAM1	20.47	3.55	2.238	9.11E-94
TUBA4A	91.201	12.54	2.768	5.18E-106
PNKD	73.18	17.22	2.026	1.46E-96
SHFM1	263.58	38.88	2.73	1.71E-112
PTPN12	39.639	8.93	2.033	4.45E-75
POR	87.19	17.98	2.216	3.68E-58
FGL2	6.92	0.72	2.203	4.66E-59
GNAI1	11.8	1.85	2.167	5.06E-79
ZFP36	207.231	34.26	2.562	4.55E-50
SRD5A3	43.84	7.67	2.371	7.76E-94
YWHAH	137.834	26.99	2.31	5.65E-92
MGAT3	7.63	1.09	2.046	3.25E-65
TPST2	18.4	132.064	-2.778	9.31E-78
RAC2	52.001	1.86	4.212	1.64E-111
LIF	17.771	0.58	3.57	1.87E-83
KRT17	295.824	0.67	7.474	1.50E-99
SPECC1	15.39	2.66	2.163	1.56E-95
FLNC	4.47	0.25	2.13	9.29E-60
TWSG1	16.91	1.75	2.703	3.88E-107
ISLR	141.925	3.04	5.145	3.67E-109
LOXL1	75.609	4.1	3.909	3.13E-113
PALLD	89.61	5.52	3.797	4.51E-100
SLC44A2	91.982	21.029	2.078	9.71E-77
KLK10	69.85	0.2	5.884	5.92E-95
KLK8	11.11	0	3.598	9.70E-53
FOXA1	3.75	0.18	2.009	9.08E-67
SNX6	37.299	7.31	2.204	1.02E-90
NEDD8	199.991	45.921	2.099	1.72E-98
KLF16	14.91	2.25	2.291	1.45E-107
SAT1	545.448	110.76	2.29	1.80E-47
LDLR	56.602	7.06	2.837	1.17E-64
PVRL2	126.027	18.59	2.697	1.55E-107
KIF1A	2.76	15.911	-2.169	7.38E-39

LSM7	118.217	25.419	2.174	6.04E-78
FCHO1	5.55	0.36	2.268	2.46E-88
MAP1S	33.59	5.55	2.401	7.90E-111
SCO2	31.491	6.67	2.083	3.55E-88
PXDN	19.79	1.47	3.073	5.16E-86
LSM4	102.493	22.239	2.155	2.20E-98
HELZ2	17.39	3.32	2.09	2.24E-80
LSP1	48.789	3.96	3.327	1.62E-78
GMFG	42.801	4.06	3.114	6.73E-85
THEMIS2	21.72	1.92	2.96	1.20E-77
SLC6A8	30.01	6.28	2.091	2.25E-42
EPS8L1	112.61	12.16	3.11	1.35E-51
SH3BGRL	84.5	17.5	2.208	6.59E-89
F12	7.81	1.02	2.125	5.88E-67
IDO1	4.72	0.13	2.34	3.96E-63
PPT1	59.482	12.05	2.212	7.26E-88
SH3BP5	15.34	2.47	2.235	1.26E-70
RFTN1	21.08	1.61	3.081	8.93E-98
SLC6A6	23.35	1.26	3.429	8.61E-109
NAPSB	13.51	0.62	3.163	3.08E-70
PDLIM4	42.591	2.2	3.768	1.90E-102
GFPT2	13.66	1.76	2.409	4.48E-49
RAMP2	29.25	5.38	2.245	4.24E-74
TMEM204	29.41	5.62	2.2	2.88E-65
IL13RA1	45.509	7.25	2.495	3.16E-108
TNS4	20.559	0.06	4.346	7.04E-86
TOP2A	12.43	0.18	3.509	3.73E-120
RARA	33.77	4.86	2.569	1.67E-102
RHPN2	25.871	3.34	2.63	3.63E-72
LGALS3	513.421	31.78	3.972	2.85E-96
PODNL1	11.33	0.46	3.078	1.20E-86
MATN3	13.46	0.17	3.627	1.70E-84
PPARG	20.141	0.95	3.438	6.64E-97
TRIM22	29.1	5.55	2.2	2.28E-49
PTPRE	8.44	0.79	2.399	2.00E-101
RAN	373.664	74.31	2.315	4.33E-87
SERPINF1	244.636	59.829	2.014	4.93E-37
JCHAIN	85.07	1.57	5.066	4.12E-61
ITGB4	207.475	7.11	4.684	1.49E-100
TRIM47	54.018	7.77	2.649	4.07E-67
XAF1	21.64	3.33	2.386	5.07E-47
MATN2	22.98	3.62	2.376	5.20E-70
PCNA	54.871	12.45	2.054	2.66E-91
NXT1	21.51	4.4	2.06	4.57E-106
NES	12.86	1.91	2.252	2.86E-74
RAB25	155.545	27.05	2.481	2.17E-65
SYT11	6.93	0.69	2.23	1.27E-73

VSTM2L	20.46	0.71	3.65	2.45E-74
POMP	84.529	16.81	2.264	1.45E-115
HMGB1P5	52.691	11.19	2.139	3.39E-80
EPSTI1	20.031	0.64	3.681	2.94E-104
POSTN	148.117	0.89	6.302	3.93E-121
RNF128	28.259	4.92	2.305	9.18E-67
SLC39A11	25.93	5.73	2.001	6.65E-100
EPHB2	7.95	1.03	2.14	2.66E-66
RARRES3	111.917	3.94	4.515	1.05E-105
HRASLS2	4.88	0	2.556	1.03E-61
MYH11	22.811	3.42	2.43	3.30E-42
GIMAP4	15.57	2.85	2.106	1.33E-61
MICAL2	28.521	4.96	2.308	1.45E-82
TTC9	5.97	0.46	2.255	6.06E-86
LOXL2	23.88	2.19	2.963	1.85E-84
IRAK2	6.1	0.57	2.177	5.20E-80
TSPAN2	7.9	0.59	2.485	1.00E-70
PTGFRN	54.371	8.34	2.568	6.26E-84
RSAD2	6.91	0.7	2.218	5.66E-67
LDHA	511.751	70.599	2.84	1.85E-69
SAA2	14.79	2.05	2.372	5.28E-26
ERN2	35.041	0.23	4.873	8.54E-87
GRP	6.51	0.2	2.646	4.74E-44
SPX	0.12	14.59	-3.799	7.06E-118
FAM127A	95.299	21.69	2.085	1.23E-79
PHC2	100.663	21.05	2.205	1.66E-83
FADS2	21.561	3.27	2.402	6.29E-64
TCN1	101.504	0.69	5.922	1.70E-83
UBAC2	61.371	14.15	2.042	4.31E-101
KDELC1	6.12	0.73	2.041	8.17E-89
ETS1	35.49	4.45	2.743	6.07E-85
NREP	46.122	2.65	3.69	7.20E-117
OSTF1	41.63	7.02	2.41	1.98E-107
TMEM2	49.121	9.45	2.262	4.43E-73
GOLM1	130.779	14.39	3.098	6.87E-100
PSAT1	4.41	21.58	-2.061	7.39E-51
HAVCR2	7.66	0.57	2.464	3.53E-71
SDS	6.69	0.23	2.644	4.52E-66
OASL	11.54	0.13	3.472	3.25E-95
TES	44.581	9	2.188	1.04E-81
MDFIC	11.9	1.45	2.397	8.27E-78
NT5E	16.29	0.57	3.461	8.40E-113
PRR5L	11.69	0.6	2.988	4.16E-105
LMO2	13.42	2.33	2.114	6.23E-73
GLS2	1.55	20.21	-3.056	1.02E-81
TROAP	6.49	0.3	2.526	1.48E-92
KRT7	517.924	29.1	4.108	6.49E-63

GNS	48.87	8.91	2.331	4.58E-99
KCNK1	30.96	1.77	3.528	1.06E-108
NTPCR	48.04	11.21	2.006	8.24E-93
STX6	13.01	1.59	2.435	1.31E-126
NPL	9.65	1.34	2.186	7.97E-77
LAMC1	51.161	8.77	2.417	1.76E-78
ITM2C	123.493	19.601	2.595	6.91E-95
SERPINE2	32.69	1.63	3.679	2.20E-87
WNT10A	5.06	0.03	2.557	5.67E-69
PLXNC1	5.56	0.55	2.081	6.34E-75
VILL	51.951	1.51	4.399	8.64E-105
FLNB	172.373	38.539	2.133	2.08E-58
LRCH1	10.65	1.45	2.249	9.67E-106
LMO7	88.738	13.25	2.655	3.60E-89
SCEL	5.29	0.07	2.555	9.53E-65
ITM2B	886.943	142.468	2.63	6.46E-99
LCP1	33.261	2.08	3.476	3.70E-79
GPNMB	89.393	4.46	4.049	2.00E-82
RAC1	288.314	42.949	2.719	3.29E-109
TTYH3	46.859	3.37	3.453	4.81E-107
LIMD2	19.8	3.11	2.339	1.64E-60
RTP4	11.8	0.79	2.838	6.57E-102
MRPL47	34.401	6.25	2.288	1.82E-119
GALNT5	8.6	0.01	3.249	1.36E-93
SKIL	18.43	2.2	2.602	4.72E-93
HLX	11.89	1.75	2.229	7.69E-75
IL1RN	34.51	0.39	4.675	5.08E-101
LRRC8A	46.52	6.59	2.646	7.01E-92
TXN	274.05	44.649	2.591	5.84E-99
KLF4	27.41	3.13	2.782	3.84E-87
PIM1	33.779	6.35	2.242	1.08E-52
TFAP2A	5.75	0.14	2.566	1.64E-85
HMGA1	149.075	11.51	3.585	3.31E-110
IER3	133.972	25.591	2.344	7.75E-44
FGFBP1	4.09	0.11	2.197	6.07E-53
SYTL2	27.1	2.02	3.218	5.87E-96
LRRC32	59.05	6.01	3.099	2.90E-95
SULF1	123.356	1.14	5.861	6.95E-118
SDCBP	131.279	24.55	2.372	3.92E-88
SORL1	13.26	2.07	2.216	1.24E-79
TMPRSS4	136.305	0.08	6.99	1.29E-129
MMP7	794.938	20.11	5.237	4.58E-61
YAP1	30.63	5.65	2.25	2.23E-68
TRIM29	99.85	0.75	5.849	1.18E-94
THBS1	87.111	12.01	2.76	2.46E-38
NUSAP1	10.83	0.61	2.877	3.03E-104
ITGA11	9.23	0.34	2.933	1.35E-77

PLCB2	12.55	2.05	2.151	2.63E-42
STRA6	14.03	0.09	3.785	5.21E-97
GCHFR	30.969	5.1	2.39	6.12E-87
IFI44L	9.02	0.48	2.759	2.73E-57
IFI44	30.909	3.54	2.813	4.89E-46
IFT172	53.69	9.17	2.427	3.60E-80
TRIM54	3.87	0.14	2.095	2.58E-55
MYOF	54.33	3.02	3.783	1.88E-106
ENTPD1	15.67	1.06	3.017	1.07E-102
ITGAV	39.829	6.47	2.45	1.08E-72
SLC40A1	71.639	14.46	2.232	5.99E-64
GUCA1C	0	12.26	-3.729	3.63E-93
TMOD3	17.99	3	2.247	2.76E-87
SHF	8.32	0.92	2.279	8.77E-98
SEMA7A	20.43	0.37	3.967	2.03E-95
RASGEF1B	7.08	0.61	2.327	8.64E-105
NAAA	16.61	3.06	2.117	4.33E-85
G3BP2	43.251	10.02	2.006	5.48E-87
11-Sep	40.201	6.8	2.401	1.12E-84
SHROOM3	19.2	1.88	2.81	3.67E-97
LEF1	17.74	0.31	3.838	2.73E-111
PPP3CA	17.95	2.86	2.296	2.86E-113
RGS3	55.008	11.04	2.218	1.80E-84
PARVG	11.54	1.96	2.083	8.72E-42
ERP27	5.06	131.789	-4.454	1.02E-67
GLIPR1	16	1.32	2.873	4.47E-75
PHLDA1	28.291	3.8	2.609	5.24E-86
LUM	974.352	25.751	5.188	2.49E-99
SNRPF	31.34	6.85	2.043	1.10E-109
SLC39A5	6.1	174.876	-4.631	5.87E-83
LPAR6	20.129	3.2	2.331	6.97E-78
RHOF	18.87	1.43	3.032	2.26E-96
FRMD6	15.03	1.64	2.602	3.65E-77
GCNT3	61.362	0.32	5.562	3.45E-106
GTF2A2	89.759	17.579	2.288	9.03E-111
SRP14	337.419	79.362	2.074	5.36E-87
TSPAN3	340.78	46.449	2.849	3.97E-101
PML	52.399	7.26	2.693	1.18E-101
PCSK6	7.58	1.12	2.017	1.94E-66
HAPLN3	15.94	0.66	3.351	8.08E-93
MFGE8	114.761	19.38	2.506	9.62E-65
IQGAP1	102.593	20.45	2.272	1.83E-89
ITGAX	10.05	1.2	2.329	4.57E-47
TGFB1I1	36.481	3.75	2.98	1.44E-88
IGSF6	5.13	0.29	2.248	1.85E-70
NLRC5	16.511	2.88	2.174	1.04E-62
MAP1LC3B	49.98	11.59	2.018	4.65E-90

IRF8	13.3	1.17	2.72	1.08E-70
KSR1	13.23	56.032	-2.003	1.56E-57
SKAP1	12.31	1.77	2.265	7.34E-63
GALNT1	28.23	6.21	2.019	5.54E-91
TMC6	46.32	8.67	2.291	2.02E-57
SLC16A3	101.293	1.89	5.146	3.76E-120
SECTM1	11.35	1.2	2.489	2.87E-62
PMAIP1	4.92	0.3	2.187	3.18E-87
ERBB2	68.09	16.14	2.011	1.22E-52
IGFBP4	399.845	51.99	2.919	5.97E-78
FKBP10	66.87	7.66	2.97	2.14E-86
PPAP2C	108.352	13.81	2.884	2.52E-62
VAV1	8.25	0.45	2.673	5.39E-82
IFITM3	974.149	176.093	2.461	6.13E-51
SOD1	235.502	42.801	2.433	5.51E-103
SAE1	53.039	10.96	2.176	2.27E-106
TM4SF5	19.59	0.16	4.15	3.39E-88
RCN3	54.518	4.73	3.276	3.05E-85
MMEL1	11.81	0.98	2.694	2.87E-62
PADI1	7.34	0.02	3.031	1.45E-49
EPHA2	49.439	3.77	3.403	2.62E-77
PGD	72.218	12.82	2.405	2.19E-100
SH3BGRL3	316.946	46.15	2.753	3.93E-94
IL22RA1	12.11	64.4	-2.319	1.01E-51
EVA1B	22.95	4.34	2.165	2.95E-57
HSPG2	127.937	16.62	2.871	1.53E-85
PRKACB	19.09	3.01	2.325	2.96E-85
TINAGL1	101.75	14.96	2.687	5.65E-68
KIF2C	4.56	0.19	2.224	2.01E-103
LMO4	72.458	11.05	2.608	1.63E-80
PSMA5	64.641	14.99	2.037	7.78E-96
TMCO1	105.843	19.81	2.36	3.93E-114
MGST3	178.267	40.559	2.109	1.21E-91
PVRL4	20.72	1.66	3.029	3.29E-68
FCGR2A	29.25	2.46	3.128	8.55E-67
NUF2	3.63	0.11	2.06	1.49E-102
RGS5	38.961	6.12	2.489	3.28E-67
PFDN2	109.212	16.66	2.642	5.16E-113
HDGF	195.998	38.191	2.33	3.32E-93
RGS16	12.79	2.25	2.085	7.58E-53
HMCN1	5.5	0.29	2.333	5.27E-52
TPM3	276.224	39.78	2.765	3.67E-105
SLC39A1	103.838	17.56	2.498	1.86E-96
SCCPDH	34.279	6.6	2.215	2.27E-106
MBOAT2	19.51	0.96	3.387	5.40E-113
PLEKHA6	20.559	3.46	2.273	2.60E-82
REG3G	7.8	605.254	-6.106	1.48E-38

SNRPG	108.052	23.1	2.178	1.48E-100
MALL	64.368	1.35	4.798	4.68E-118
RALB	49.841	7.16	2.639	1.04E-113
VGLL4	41.76	9.39	2.041	2.89E-78
OSBPL10	8.49	0.55	2.614	8.42E-113
NR1I2	3.72	0.13	2.062	5.85E-49
NCEH1	11.57	1.48	2.342	4.52E-84
TMEM44	15.899	2.43	2.301	1.96E-94
MUC4	10.2	0.36	3.042	2.57E-53
OCIAD2	215.582	14.55	3.8	9.63E-134
PLAC8	65.721	0.5	5.475	1.52E-116
SNCA	5.96	0.74	2	6.49E-58
SFRP2	351.114	5.74	5.707	1.62E-82
PDGFC	25.96	4.48	2.299	7.38E-55
GZMA	7.96	0.32	2.763	3.03E-79
PAM	144.908	26.591	2.403	1.55E-78
RNF145	45.981	6.59	2.63	2.87E-101
ZMAT2	68.559	14.89	2.13	6.64E-104
PLA2G7	5.54	0.11	2.559	3.87E-74
TNFRSF21	71.421	4.44	3.735	1.38E-115
PPP1R18	60.951	6.07	3.131	2.12E-88
NFKBIE	21.31	2.48	2.681	2.12E-98
TPBG	17.08	2.9	2.213	1.03E-74
SLC22A3	6.21	0.19	2.599	3.10E-82
IGFBP3	260.113	4.18	5.656	2.87E-122
TRIM50	0.16	11.04	-3.376	7.08E-108
SH3KBP1	26.44	1.28	3.589	7.12E-139
MSN	135.213	11.97	3.393	1.99E-89
SNX12	35.631	7.69	2.076	1.56E-111
IL2RG	61.422	0.75	5.157	4.52E-117
NSDHL	19.721	4.16	2.006	1.77E-113
ZNF185	11.91	1.08	2.634	1.56E-87
MAL2	118.767	11.55	3.255	3.90E-92
FAM83A	7.8	0.02	3.109	1.08E-58
SLC39A4	77.288	10.8	2.73	6.23E-73
NAPRT	92.558	16.14	2.448	4.29E-49
STOM	73.16	11.1	2.616	3.27E-84
GSN	407.54	46.099	3.117	5.96E-100
PTGES	18.41	0.42	3.773	2.72E-93
LCN2	1033.769	14.86	6.028	1.18E-71
MSRB2	38.08	3.86	3.007	3.91E-122
ZEB1	11.79	1.59	2.304	1.46E-69
MKI67	7.93	0.09	3.034	3.08E-111
GSTO1	151.239	29.931	2.299	2.21E-99
ITPRIP	14.09	1.71	2.477	1.10E-89
RGS10	44.811	2.03	3.918	6.77E-124
SYT8	42.121	7.73	2.304	1.29E-20

SERPING1	450.161	110.506	2.017	1.16E-36
SLC43A1	10.15	78.248	-2.829	7.41E-88
SERPINH1	191.114	11.2	3.977	1.73E-105
P4HA3	4.2	0.16	2.164	2.55E-62
ST14	130.119	20.78	2.59	9.76E-76
FEZ1	10.69	1.3	2.346	5.54E-85
MPZL2	43.849	6.19	2.641	2.77E-64
TAGLN	844.877	72.368	3.527	2.57E-71
KIAA1755	5.4	0.47	2.122	7.90E-76
GPHA2	0.2	21.029	-4.198	4.35E-63
FERMT3	28.271	2.39	3.11	1.57E-84
PLCB3	20.7	3.35	2.319	3.13E-88
PPP4C	103.4	19.71	2.334	3.97E-105
ITGB1	364.986	36.41	3.29	3.15E-100
FCGR1A	16.151	1.05	3.065	7.13E-79
HNMT	53.341	10.29	2.267	1.41E-69
PDCD4	82.761	357.747	-2.099	2.26E-53
VEGFC	6.1	0.53	2.214	8.98E-87
PRSS23	60.951	5.85	3.177	2.41E-97
IL18	42.32	4.68	2.931	6.39E-81
UBC	1175.451	228.998	2.355	1.92E-84
MAT1A	0.56	8.91	-2.667	2.39E-99
NDUFC2	198.445	46.869	2.059	1.84E-101
EPS8	38.641	5.74	2.556	1.24E-78
MFSD6	22.62	4.34	2.145	1.80E-98
TMEM45B	23.271	0.43	4.085	1.76E-110
SPOCK1	12.46	1.86	2.235	3.69E-48
XRCC4	7.81	1.03	2.118	7.12E-96
TMEM123	94.549	19.9	2.193	5.35E-77
GJA1	36.569	3.11	3.192	2.53E-83
PELO	14.1	2.75	2.01	1.32E-110
SLC16A12	0.72	7.83	-2.36	1.72E-52
GGPS1	25.701	5.52	2.034	1.70E-110
MR1	13.1	1.26	2.641	1.42E-113
PLA2R1	5.12	0.33	2.202	2.10E-97
RBMS1	26.99	4.46	2.358	3.61E-75
FAM49B	36.46	6.9	2.245	1.13E-91
THY1	179.606	4.94	4.926	2.06E-102
TDH	0.1	5.17	-2.488	1.37E-84
SH3RF1	13.3	2.32	2.107	7.42E-90
MAGED4	6.55	0.62	2.22	2.82E-56
PDLIM3	38.81	5.65	2.582	2.55E-52
TCEB1	67.949	15.14	2.095	1.88E-101
LY96	18.56	1.2	3.152	2.78E-87
WNT7A	3.07	0.01	2.011	3.05E-51
FGD5	13.12	1.24	2.656	5.25E-94
VOPP1	63.092	8.01	2.831	1.46E-125

PROM2	29.14	5.66	2.178	8.20E-32
PI4K2A	13.15	2.17	2.158	1.43E-110
MARVELD1	36.959	2.63	3.386	5.13E-113
SAMSN1	6.01	0.58	2.149	1.65E-59
RHOC	435.732	47.98	3.156	2.66E-101
SLC16A1	12.01	0.77	2.878	2.34E-110
SLC7A7	28.919	2.43	3.125	2.10E-94
VSIG4	20.77	3.97	2.131	1.31E-34
FZD7	7.09	0.54	2.393	2.02E-88
SLA	10.51	1.31	2.317	1.53E-50
MCU	28.259	3.79	2.611	1.80E-99
FUT6	12.42	0.98	2.761	4.22E-72
PCDH1	48.08	5.15	2.996	7.56E-93
HKDC1	16.71	2.39	2.385	1.37E-54
HK1	44.811	4.47	3.066	8.91E-114
UBE2L6	80.258	8.32	3.124	3.13E-98
NPTN	93.422	22.529	2.005	1.82E-76
FBXO32	39.59	3.28	3.245	1.04E-88
TMEM171	5	0.18	2.346	7.63E-84
NECAP2	53.619	9.57	2.369	5.83E-97
MMP14	226.944	13.27	3.998	9.56E-94
TMED6	3.29	47.408	-3.496	4.80E-59
MYO1E	70.869	5.69	3.425	1.03E-111
ETS2	55.05	6.81	2.843	2.68E-83
TSPAN18	6.31	0.55	2.238	5.13E-79
MX1	38.051	3.77	3.033	3.41E-77
PSMG3	50.348	9.82	2.247	7.97E-100
FAM213B	36.121	3.32	3.103	2.96E-111
SLC30A2	2.2	31.089	-3.326	3.77E-60
MRPL17	61.55	13.88	2.072	6.34E-112
NCK1	23.46	5.02	2.023	3.84E-80
GPR153	7.97	0.49	2.59	1.19E-98
RHBDL2	8.78	0.21	3.015	8.71E-105
HIST1H2BD	22.38	2.94	2.569	5.62E-54
NCF1	6.53	0.59	2.244	1.22E-53
TAGLN2	788.244	97.998	2.995	3.95E-89
SLAMF8	9.61	0.61	2.72	1.14E-77
SLC45A3	12.32	0.44	3.209	5.35E-99
NBL1	256.906	8.03	4.836	4.20E-132
F11R	73.832	15.39	2.191	3.37E-74
FCER1G	90.421	7.83	3.372	1.70E-71
IFNAR2	21.84	3.64	2.299	1.20E-97
IFNGR2	100.551	11.34	3.041	2.12E-113
LAD1	167.369	21.021	2.935	2.67E-64
STC1	12.77	1.04	2.755	1.06E-75
RUNX1	29.311	1.32	3.708	3.42E-108
PTMS	347.651	62.621	2.454	6.37E-85

HK2	19.3	0.78	3.512	1.41E-99
SPON2	95.14	9.66	3.173	1.70E-76
TPPP3	46.179	5.34	2.896	2.59E-70
ZYX	152.979	30.73	2.279	1.54E-69
TFF3	201.396	3.43	5.514	2.34E-74
TFF2	138.169	0.51	6.526	1.23E-73
TFF1	481.702	0.29	8.548	6.92E-86
TMPRSS3	26.811	2.6	2.95	1.54E-75
SLC37A1	18.77	2.62	2.449	3.24E-102
CBS	2.92	66.921	-4.115	1.38E-98
G6PD	26.999	5.84	2.033	2.36E-93
ITGB2	61.46	3.25	3.877	3.39E-85
S100B	11.07	1.5	2.271	2.56E-53
LMNA	319.86	33.291	3.226	1.22E-113
LY6E	395.764	19.74	4.258	2.24E-98
ORAI2	11.71	2.06	2.054	9.95E-87
MGAT4B	165.891	31.15	2.376	1.06E-82
PSMC2	46.562	10.83	2.007	1.33E-99
NPHS1	0.42	11.61	-3.151	1.86E-120
PLXDC1	12.87	1.92	2.248	3.47E-70
ITGA5	57.229	8.36	2.637	5.15E-68
ZNF385A	13.74	1.33	2.661	1.57E-93
PLCD3	41.581	2.52	3.597	3.89E-102
FMNL3	9.25	1.13	2.267	1.48E-82
RACGAP1	8.79	0.78	2.459	2.75E-115
SPC24	3.9	0.18	2.054	5.89E-97
TNFSF13	51.118	11.15	2.101	9.09E-72
FLYWCH2	27.779	5.19	2.217	4.46E-106
ZG16B	42.449	0.41	4.946	2.40E-87
RPS6KA4	27.959	5.62	2.129	1.19E-89
PDZK1IP1	217.82	35.68	2.577	1.91E-37
FBLIM1	60.748	13.24	2.116	5.48E-74
PDPN	22.201	1.92	2.99	1.21E-71
LAPTM5	124.507	6.22	4.12	1.53E-89
SDC3	19.43	2.53	2.533	4.65E-92
MXRA8	160.096	9.95	3.879	8.23E-91
FAM102B	7.3	1.07	2.003	7.32E-83
HENMT1	7.94	0.52	2.556	1.47E-108
GBP2	71.14	3.58	3.977	3.48E-110
GBP4	11.51	0.75	2.838	1.37E-99
VCAM1	20.941	3.19	2.389	2.90E-44
PEA15	117.759	16.31	2.778	6.44E-103
OLFML2B	32.561	0.53	4.455	3.68E-112
IER5	10.78	1.94	2.002	3.69E-86
KIF26B	4.84	0.1	2.408	8.30E-94
PM20D1	0.39	27.429	-4.354	5.53E-75
PKDCC	63.672	13.6	2.147	4.03E-51

PIGR	104.047	3.5	4.545	4.66E-59
FRZB	10.35	0.81	2.649	1.16E-73
H3F3A	614.853	122.93	2.313	1.10E-89
SGCB	15.88	2.14	2.426	8.34E-107
SGPP2	19.251	1.03	3.318	5.06E-101
PDLIM5	34.621	7.29	2.103	3.03E-91
SCNM1	43.419	9.95	2.02	7.78E-97
S100A11	2034.84	81.509	4.625	8.47E-100
S100A9	65.94	14.44	2.116	2.34E-26
TGFA	12.56	1.94	2.205	1.31E-68
PBXIP1	74.299	13.13	2.414	7.23E-102
IGFN1	0.3	8.2	-2.823	1.61E-91
FSTL1	100.642	8.02	3.494	4.85E-85
IGFBP7	779.172	97.092	2.992	2.36E-81
IHH	6.03	0.02	2.785	5.00E-62
FBLN2	31.34	2.14	3.364	9.78E-80
PRKCI	16.42	3.35	2.002	1.72E-84
MNDA	6.68	0.62	2.245	2.79E-54
IFI16	83.87	6.29	3.541	1.90E-94
TIPARP	16.19	3.05	2.086	6.33E-83
ZDHHC3	43.03	8.84	2.162	2.17E-94
KLF15	2.3	18.64	-2.573	7.15E-84
LIPH	25.189	1.5	3.389	4.06E-91
RPN1	176.974	41.191	2.077	4.57E-98
RPL39L	10.38	0.76	2.693	9.80E-82
MFI2	18.43	2.17	2.616	2.31E-64
S100P	525.737	0.28	8.685	5.85E-109
H2AFZ	168.265	32.199	2.35	9.37E-102
SLC9B2	13.86	0.95	2.93	6.56E-90
SHISA5	135.363	23.669	2.467	2.85E-98
MST1R	52.778	3.39	3.615	5.12E-77
POC1A	4.48	0.34	2.032	3.00E-112
HMGB2	76.378	10.31	2.774	9.21E-98
MAD2L1	7.4	0.44	2.544	6.99E-117
HPGD	18.85	0.34	3.889	2.31E-98
FAM198B	19.76	2.27	2.666	5.36E-82
ITGA2	16.67	0.38	3.679	1.17E-114
F2RL2	5.48	0.24	2.386	2.74E-71
F2RL1	30.271	5.05	2.37	8.89E-71
SPINK1	1192.108	11822.11	-3.309	5.50E-38
ESM1	6.19	0.12	2.683	1.34E-84
GPX8	24.8	2.1	3.057	9.62E-99
SERINC5	12.63	2.35	2.025	1.48E-86
ERAP2	12.28	0.96	2.76	5.33E-43
FOXQ1	10.31	0.59	2.831	1.97E-79
TMEM200A	7.57	0.3	2.721	4.18E-85
GALNT10	23.11	3.59	2.393	2.06E-98

PTTG1	26.139	0.63	4.057	6.41E-139
STEAP1	22.511	2.35	2.811	5.44E-85
SHH	4.38	0.04	2.371	6.35E-75
FNDC1	12.98	0.34	3.383	1.88E-82
RAD21	48.421	10.29	2.13	4.41E-94
TNFRSF11B	9	0.44	2.796	1.77E-64
MICALL2	48.351	9.81	2.191	3.97E-47
YWHAZ	382.84	45.959	3.031	2.22E-105
GEM	30.181	1.35	3.73	4.65E-89
LETM2	8.11	1.18	2.063	1.37E-70
TRPV6	8.21	37.801	-2.075	4.49E-36
MELK	4.43	0.12	2.277	8.91E-92
SPTSSA	22.279	4.47	2.089	1.24E-94
FOLR2	15.35	1.4	2.768	3.55E-66
GJB2	33.651	0.38	4.65	6.96E-91
REEP3	18.33	2.65	2.405	3.48E-99
VSTM4	4.56	0.39	2	8.74E-79
PRAP1	10.84	0.09	3.441	6.73E-64
IFI27	879.536	12.63	6.014	4.13E-123
HTRA1	177.785	17.94	3.239	5.86E-92
SPRED1	9.21	1.29	2.157	6.67E-98
JAM3	16.69	2.41	2.375	2.26E-82
RAB8B	9.98	1.34	2.23	6.60E-92
IKBIP	10.82	1.63	2.168	3.03E-96
PPP1R14D	6.57	0.09	2.796	4.26E-77
SPINT1	143.997	31.321	2.165	1.68E-65
FBN1	56.578	2.7	3.96	2.50E-90
WBP1L	27.99	4.93	2.289	6.17E-100
SERPINB8	9.4	1.51	2.051	5.55E-84
RIC3	1.96	14.47	-2.386	7.16E-70
MFAP4	71.56	11.19	2.573	7.42E-47
HDGFRP3	14.99	2.19	2.326	6.25E-72
RRAD	6.7	0.88	2.034	6.54E-51
FAM96B	127.229	28.44	2.123	2.68E-98
MMP10	3.33	0.05	2.044	3.16E-50
NNMT	422.907	24.03	4.082	3.17E-86
SLFN5	7.32	0.98	2.071	1.33E-77
KIAA0101	17.54	0.89	3.294	1.54E-108
PLK1	11.27	0.71	2.843	1.12E-105
MYO1A	4.91	0.09	2.439	1.40E-51
NAB2	17.111	2.4	2.413	9.96E-92
YWHAB	131.515	20.639	2.614	1.80E-112
GREM1	29.4	0.59	4.257	2.83E-70
MS4A7	13.33	2.11	2.204	6.73E-48
MS4A8	7.23	0.64	2.327	7.81E-46
EVA1C	33.4	6.63	2.173	5.04E-79
MEI1	6.58	0.33	2.511	6.18E-84

PHB	122.734	29.761	2.008	6.35E-101
SNRPD1	81.69	10.84	2.804	1.05E-123
TMEM92	21.63	0.36	4.057	4.68E-104
FAM102A	62.422	13.53	2.126	5.83E-76
PRRX2	6.84	0.28	2.615	3.06E-80
UGT1A6	3.54	0.03	2.14	2.01E-59
IGF2	16.2	2.27	2.395	1.75E-31
TPM4	417.056	28.01	3.849	1.61E-108
RAB8A	35.579	7.51	2.104	2.03E-101
GPX4	349.318	54.541	2.657	5.82E-104
TUBA1A	164.073	16.06	3.274	6.35E-82
TUBA1C	212.526	21.52	3.245	1.22E-90
SERTAD3	31.51	3.33	2.908	1.29E-121
LAIR1	19.2	2.04	2.732	6.35E-56
SPINT2	622.184	103.593	2.575	2.20E-86
PSCA	103.342	0.25	6.383	8.98E-69
LY6D	8.04	0.28	2.82	7.84E-37
SLC43A2	14.69	2.63	2.112	3.16E-83
KLK1	24.06	1669.151	-6.058	7.18E-71
KLK6	24.96	0.03	4.656	2.57E-76
IGFBP6	78.858	12.39	2.576	1.37E-56
EVPL	14.47	1.07	2.902	8.45E-74
TK1	21.98	1.95	2.962	3.66E-96
KCTD5	21.159	3.6	2.268	1.02E-115
FADD	16.82	3.02	2.148	8.69E-109
NAALADL1	7.79	1	2.136	1.54E-78
SCARA3	11.23	1.13	2.521	1.83E-71
PAFAH1B2	31.41	6.65	2.083	9.47E-114
NKIRAS2	25.679	5.45	2.048	4.04E-108
PTF1A	0.03	16.829	-4.114	1.49E-99
NT5DC2	45.37	7.37	2.47	7.82E-91
MFSD2A	7.48	0.2	2.821	3.41E-96
TAP1	56.92	6.49	2.951	1.69E-91
RHOH	4.49	0.28	2.101	5.64E-61
RAB31	42.2	1.87	3.912	4.36E-101
SERINC2	143.21	12.74	3.392	2.11E-77
LDLRAD4	7.58	0.75	2.294	3.94E-92
SLC16A4	8.91	0.39	2.834	1.62E-90
IL7R	7.61	0.67	2.366	2.33E-46
PKIG	44.891	9.76	2.093	9.14E-77
TSPAN5	6.93	0.46	2.441	1.17E-101
VAMP5	57.34	11.34	2.241	7.87E-85
INPP5D	11.2	1.26	2.433	1.85E-41
PPIC	64.061	11	2.439	4.39E-84
LGALS9	187.194	10.15	4.077	1.54E-97
PXDC1	38.791	6.42	2.423	7.46E-84
KLK7	20.15	0.05	4.332	6.66E-64

ROR2	6.04	0.58	2.156	9.41E-75
PGM2	14.94	2.94	2.016	1.78E-89
GP2	19.889	13619.63	-9.349	1.33E-75
PTAFR	9.5	0.49	2.817	5.89E-97
PTPN9	17.93	3.34	2.125	2.11E-101
RNASE6	17.67	1.64	2.822	4.21E-69
SPRR1B	5.25	0.33	2.232	5.01E-32
TM2D2	33.24	6.58	2.175	1.06E-105
PLEKHA2	12.77	1.55	2.433	1.81E-64
GPR183	9.84	0.51	2.844	1.38E-67
ZEB2	10.37	1.67	2.09	1.56E-60
NRG4	1.38	12.01	-2.451	4.67E-70
LIMS1	27.801	4.19	2.472	4.30E-83
PCDH7	8.07	0.12	3.018	2.39E-91
ROBO1	9.06	0.79	2.491	2.25E-76
MUC3A	34.929	0.77	4.343	2.60E-80
ITGAM	7.48	1.06	2.041	3.78E-54
TM4SF4	206.642	16.87	3.538	2.81E-51
TM4SF1	363.194	35.54	3.317	9.92E-65
MYO7B	8.81	0.34	2.872	2.20E-56
YWHAG	63.61	12.14	2.298	1.80E-100
SLC16A5	14.2	1.7	2.493	2.67E-67
FOS	235.453	57.539	2.014	3.20E-22
KRT8	1440.146	274.183	2.389	2.24E-57
METTL7B	12.77	0.89	2.865	2.30E-93
MZB1	12.97	0.23	3.506	2.70E-63
PFKFB3	35.041	5.88	2.389	1.62E-63
TMC7	5.03	0.2	2.329	1.19E-61
SERPINB9	7.71	0.73	2.332	5.28E-85
SMAGP	34.741	6.06	2.34	3.69E-76
TMEM133	4.65	0.39	2.023	2.31E-99
SDR16C5	16.4	0.08	4.01	1.95E-99
HTRA3	88.658	1.91	4.945	1.68E-109
TMEM43	46.96	9.94	2.132	1.74E-83
PLA2G1B	13.78	15125.549	-9.999	1.86E-68
OSCAR	5.43	0.37	2.231	8.03E-68
PAQR8	8.4	0.49	2.657	7.05E-100
PKIA	3.9	0.16	2.079	2.97E-85
FPR1	7.87	0.8	2.301	4.18E-52
FEZ2	26.921	4.86	2.252	1.94E-103
FUT3	43.131	2.81	3.534	3.78E-70
JUNB	197.744	39.061	2.311	2.84E-39
TMEM37	24.23	4.37	2.232	2.72E-62
PGAM1	160.24	29.681	2.394	1.73E-98
KRT19	1456.812	15.73	6.445	1.33E-114
KRT15	12.11	0.61	3.026	1.12E-69
HOPX	67.958	1.52	4.774	3.17E-111

RGS19	14.72	2.83	2.037	4.17E-76
LGALS4	459.206	17.03	4.674	2.65E-64
GATM	52.94	529.247	-3.297	5.48E-60
RHNO1	18.03	3.67	2.027	1.30E-109
NINJ2	11.21	0.48	3.044	2.74E-102
MLLT3	12.25	1.35	2.495	2.40E-105
RRM2	11.88	0.32	3.287	3.02E-105
PRNP	49.931	8.35	2.446	8.35E-92
PPIH	33.121	7.27	2.045	8.03E-103
REG3A	87.741	810.236	-3.192	5.31E-12
REG1B	15.98	9666.647	-9.153	6.07E-55
LRRC15	3.51	0.01	2.159	1.78E-57
ISG20	57.859	4.18	3.506	3.83E-106
TPSAB1	39.199	5.89	2.545	1.96E-53
GNG12	37.6	6.86	2.296	1.36E-84
RND1	7.36	0.69	2.306	2.10E-59
RARG	25.621	3.46	2.577	1.63E-92
MYEOV	36.39	0.92	4.283	8.77E-75
MYD88	36.37	6.15	2.386	4.02E-91
MIR4435-2H	79.511	5.46	3.64	2.74E-106
PARP14	17.48	3.52	2.032	1.19E-73
GLRX	37.859	4.77	2.752	9.78E-93
SNCG	21.81	2.24	2.816	1.88E-57
PPP1R3B	9.8	1.44	2.146	4.85E-85
TRIB1	26.58	5.55	2.074	1.31E-66
OLR1	12.49	0.41	3.258	4.23E-60
SAA1	38.47	5.44	2.616	3.42E-29
LRFN4	12	1.21	2.556	8.44E-74
PSMD1	51.839	9.44	2.34	8.50E-115
MUC13	62.769	0.97	5.017	3.66E-102
JUP	198.885	22.119	3.112	1.47E-96
NET1	58.801	12.95	2.1	9.18E-70
HOXB2	13.19	1.49	2.511	8.94E-80
3-Mar	9.96	1.6	2.076	2.00E-77
XXYLT1	7.93	1.09	2.095	3.30E-113
FAM3C2	31.811	4.47	2.585	1.48E-66
MSRB3	12.85	1.37	2.547	1.69E-71
SELP	5.69	0.64	2.028	1.48E-50
PHLDA3	56.881	8.65	2.584	3.35E-83
PODN	31.8	6.61	2.108	3.29E-41
KIAA1551	28.989	5.61	2.182	1.11E-78
UBE2C	28.789	0.26	4.563	7.52E-124
SPSB4	0.64	5.88	-2.069	3.26E-96
MARCKSL1	164.073	7.64	4.256	9.92E-124
PNLIP	12.66	16871.492	-10.271	1.86E-55
UCP2	64.128	4.4	3.592	1.95E-102
FOSL1	15.99	0.43	3.571	1.31E-87

SUGCT	16.42	0.58	3.463	1.35E-81
RMI2	7.55	0.46	2.55	3.88E-107
SFN	128.604	0.54	6.395	9.87E-121
ETV4	17.09	2.41	2.407	2.12E-66
GPX2	313.169	5.37	5.624	3.43E-94
HSD11B2	20.3	3.48	2.249	1.55E-48
LPCAT4	87.462	11.05	2.876	4.33E-82
SLCO3A1	16.429	2.12	2.482	5.26E-84
PLA2G16	110.921	15.61	2.752	4.87E-94
PRR15	21.66	1.03	3.481	6.44E-101
LMNB2	17.91	3.38	2.11	2.72E-106
MYO1D	47.1	10.41	2.076	2.22E-75
FOXL1	6.51	0.04	2.852	2.59E-88
METRNL	56.09	8.92	2.525	2.18E-72
TYMS	16.88	3.24	2.076	5.40E-85
PNMA1	30.33	2.46	3.179	5.26E-121
FUT2	27.05	3.18	2.746	8.34E-53
MUC20	57.308	7.3	2.813	9.35E-51
FIBIN	9.06	1.25	2.161	5.21E-50
RHOG	65.231	11.38	2.419	2.43E-100
HIC1	7.79	1.13	2.045	4.53E-60
SAMD9L	8.52	1.09	2.187	1.20E-72
TGIF1	68.711	12.77	2.34	3.69E-71
PTRF	95.51	9.46	3.206	6.23E-96
GBA	51.929	11.78	2.05	9.03E-111
FAM20C	37.361	7.26	2.215	3.91E-75
GRB2	54.64	11.15	2.195	6.77E-102
UBE2N	61.158	12.25	2.23	4.10E-100
ODF3B	32.94	6.1	2.257	9.51E-49
MLF1	10.52	1.5	2.204	1.36E-84
KDELC2	15.67	2.92	2.088	7.11E-88
PLEC	71.988	10.01	2.729	3.02E-85
MAF	8.55	1.15	2.151	3.00E-83
GPR35	21.07	0.55	3.832	4.52E-98
KCTD12	21.01	2.43	2.682	6.55E-83
TMEM52	1.93	85.407	-4.882	1.08E-108
RNF186	1.77	14.15	-2.451	2.91E-56
MSC	11.38	1.34	2.403	1.17E-60
FAM101A	14.21	0.1	3.789	2.57E-105
FBXO34	15.401	2.92	2.065	8.24E-93
SEPW1	431.255	74.511	2.517	3.57E-95
RCC2	47.441	7.69	2.479	2.98E-100
FUCA1	47.53	7.28	2.551	5.05E-116
HLA-DQB1	83.487	3.34	4.283	1.50E-64
VWA1	99.098	16.979	2.477	1.20E-64
PCED1B	10.13	0.91	2.543	7.49E-89
SYCN	4.07	5355.478	-10.045	1.12E-69

FOXS1	7.38	0.25	2.745	1.12E-91
MYADM	96.763	9.75	3.185	7.24E-101
GPBAR1	6.64	0.6	2.256	3.24E-72
SSC5D	10.07	0.51	2.874	1.19E-84
TMEM150B	3.93	0.15	2.1	1.30E-58
HCLS1	28.969	5.19	2.275	8.06E-49
PAK2	21.72	4.55	2.033	3.90E-96
HMHA1	23.03	4.69	2.078	7.31E-72
HIST1H2AC	30.3	3.37	2.84	1.61E-72
HIST1H2BC	5.69	0.52	2.138	7.55E-65
SHISA2	4.05	0.1	2.199	2.79E-96
ZDHHC20	26.99	3.56	2.618	1.66E-118
LINC01559	4.89	0	2.558	3.69E-60
MRPL14	52.921	8.99	2.432	6.31E-123
NQO1	235.551	2.87	5.934	3.85E-137
HIGD1A	77.961	16.77	2.152	8.39E-91
F2R	31.86	1.22	3.888	1.48E-108
MUC16	7.68	0.02	3.089	2.55E-46
PJA1	21.01	3.99	2.141	1.53E-108
HIST3H2A	10.63	1.45	2.247	6.83E-78
RAP2B	12.84	1.28	2.602	1.14E-118
FDCSP	9.14	0	3.342	1.89E-43
PHLDA2	44.68	1.03	4.492	5.96E-81
NOP10	177.33	33.4	2.374	6.30E-101
TP53TG1	33.981	5.12	2.515	6.92E-123
EXT1	32.64	6.7	2.127	1.38E-86
TSHZ2	10.04	1.03	2.443	7.21E-84
KPNA2	30.6	3.13	2.936	1.20E-121
TRAK1	31.421	4.28	2.618	5.39E-109
SKA2	22.15	4.48	2.079	6.77E-104
NTM	18.16	0.18	4.021	2.95E-74
TSKU	34.391	6.05	2.328	7.04E-71
VMO1	5.38	0.42	2.168	2.99E-82
LCK	8.98	0.35	2.886	1.00E-83
TCEAL7	4.81	0.41	2.043	6.20E-73
HMGN4	46.539	9.85	2.131	6.25E-86
GPC6	4.5	0.18	2.221	5.23E-82
TMEM119	28.24	2.17	3.205	7.27E-71
PTTG1IP	208.037	29.85	2.76	7.99E-110
15-Sep	199.424	42.78	2.195	3.39E-93
FHL3	28.019	5.85	2.083	4.93E-65
GPR132	4.3	0.16	2.192	8.58E-88
MX2	15.49	1.92	2.498	3.31E-73
HMCES	34.621	5.46	2.463	7.26E-120
UPP1	35.981	5.24	2.567	2.90E-84
LHFP	28.259	4.66	2.37	4.04E-68
TMEM50A	100.349	17.881	2.424	3.43E-105

ZNF703	26	3.26	2.664	6.36E-102
OLFML1	7.09	0.75	2.209	5.60E-68
KIRREL	12.23	1.45	2.433	3.69E-74
IQGAP3	4.8	0.07	2.438	5.90E-91
PTP4A2	151.964	33.52	2.148	2.82E-85
PPP1R2	32.329	6.5	2.152	2.39E-99
TACSTD2	168.826	24.96	2.71	7.30E-35
PKP3	62.76	13.01	2.186	1.57E-57
PROS1	16.47	2.09	2.499	5.88E-91
SOCS3	50.31	7.05	2.672	1.53E-51
LPAR5	6.36	0.21	2.605	1.40E-107
TMEM173	60.141	5.09	3.328	1.08E-106
9-Sep	144.787	14.77	3.209	3.78E-113
ZBTB7C	3.99	0.23	2.02	1.26E-78
SUMO3	71.878	15.05	2.183	3.57E-103
FMNL1	20.841	3.03	2.438	9.69E-63
USP18	8.21	1.11	2.126	2.66E-92
MAFF	30.34	3.12	2.927	6.82E-89
SLC24A3	5.93	0.27	2.448	9.00E-101
IFITM2	452.539	69.932	2.677	1.30E-57
TNFAIP2	126.851	14.63	3.032	3.86E-59
WBP5	73.93	16.7	2.082	1.90E-82
UBALD2	80.61	16.01	2.262	1.82E-83
TCN2	26.009	4.32	2.344	6.69E-93
TNFAIP8L1	7.28	0.99	2.057	5.07E-103
SMYD3	20.75	2.67	2.567	6.53E-129
KRT6B	8.37	0.17	3.002	3.46E-53
MUC1	777.985	97.342	2.986	1.40E-41
IRF7	28.59	5.9	2.1	3.85E-46
PDIA2	5.19	671.2	-6.763	2.51E-80
IFIT1	16.79	1.68	2.731	1.47E-63
MORF4L1	216.061	51.599	2.045	1.27E-77
EVI2B	10.6	0.75	2.729	1.73E-66
TRIM69	15.1	1.62	2.619	9.72E-100
IFITM1	223.123	28.489	2.926	1.00E-68
ZNF267	8.18	1.1	2.128	2.75E-108
LEMD1	15.11	0.05	3.939	6.98E-81
NDUFA13	320.015	62.171	2.345	2.97E-107
SAPCD2	3.75	0.12	2.084	1.32E-93
THBS2	91.512	1.61	5.147	1.08E-102
GNG2	7.59	1.03	2.081	4.45E-61
10-Sep	27.07	5.44	2.124	1.68E-82
LILRB4	10.82	0.59	2.894	4.68E-71
KRT16	28.999	0.15	4.705	1.86E-76
ESPN	6.5	0.23	2.608	2.67E-63
PNLIPRP1	3	3098.812	-9.598	3.37E-84
GCNT1	6.02	0.48	2.246	3.59E-80

SESTD1	21.88	3.65	2.299	4.35E-83
FPR3	7.74	0.48	2.562	3.51E-65
ISG15	113.322	4.35	4.417	1.53E-93
TMSB4XP8	14.58	0	3.962	4.64E-28
SPRY4	13.05	1.58	2.445	2.83E-104
TRPV2	17.92	2.64	2.378	4.93E-73
FANCA	9.56	1.28	2.212	1.08E-82
TPRG1	4.51	0.33	2.051	1.68E-62
UBQLN2	16.47	3.06	2.105	6.59E-106
WNT7B	5.75	0.16	2.541	5.74E-85
TUBB4B	197.156	37.809	2.352	1.39E-91
PLSCR1	85.828	17.66	2.218	2.71E-62
SLC38A3	1.37	17.91	-2.996	1.86E-67
SELL	6.68	0.26	2.608	7.69E-62
IER5L	12.77	1.46	2.485	6.71E-63
SUMO2	267.148	50.369	2.384	1.18E-101
S100A16	384.622	13.74	4.709	2.41E-119
SMIM15	33.3	6.75	2.146	1.02E-103
PRELP	24.54	4.18	2.302	3.21E-44
FAM26F	7.96	0.53	2.55	3.27E-73
GJB3	28.07	0.1	4.724	1.53E-113
LITAF	117.197	14.72	2.911	1.09E-96
HN1	233.778	7.69	4.756	4.84E-131
S100A13	203.233	29.84	2.727	4.43E-95
GJB5	4.7	0.08	2.4	3.35E-52
FAM150B	3.17	25.63	-2.675	5.40E-53
S100A14	189.505	5.27	4.925	5.25E-83
RP6-149D17	0	51.749	-5.721	3.24E-26
HMGB1	272.006	65.181	2.044	1.41E-85
MMP23B	11.99	1.54	2.354	1.62E-67
GJB4	4.01	0.01	2.31	5.70E-71
IL1RAP	8.38	0.82	2.366	8.90E-81
HLA-DRB1	290.178	10.67	4.641	9.21E-95
SPATS2L	139.352	15.75	3.067	5.52E-103
S100A4	313.038	17.6	4.078	3.24E-84
TUBB	280.061	50.101	2.459	1.44E-86
SFTA2	64.128	1.54	4.68	1.22E-76
PPIA	1549.728	365.974	2.079	6.43E-72
MYL6B	47.5	7.76	2.469	6.28E-105
SPTSSB	5.07	0.22	2.315	1.87E-63
SULF2	108.06	3.55	4.583	4.62E-124
MMP1	59.999	0.15	5.729	2.98E-84
HRH1	5.36	0.11	2.518	1.08E-115
HSH2D	14.62	2.49	2.162	5.78E-73
HLA-DQA1	57.281	1.41	4.596	1.85E-87
GM2A	25.149	3.57	2.517	8.40E-105
S100A2	27.469	0.59	4.162	1.03E-67

LAMB3	172.278	1.13	6.346	4.50E-120
PDLIM7	127.027	8.39	3.769	5.75E-96
FLNA	518.176	66.509	2.943	4.85E-66
FUT11	16.151	3.27	2.006	4.84E-94
SERTAD1	19.821	2.14	2.729	1.78E-114
GAL3ST4	6.36	0.44	2.354	1.13E-81
HIST1H4J	6.68	0.36	2.498	3.92E-87
SERPINA1	830.593	42.511	4.256	9.96E-70
TPSB2	36.64	5.56	2.521	4.30E-45
PELI1	17.43	3.22	2.127	1.08E-79
HNRNPAB	117.652	22.389	2.343	2.84E-106
SPN	3.89	0.22	2.003	1.21E-67
MFAP5	26.29	1.03	3.749	5.06E-73
MPEG1	6.2	0.65	2.126	2.11E-54
SERPINB2	4.15	0.21	2.09	5.48E-45
SPTAN1	127.698	30.89	2.013	2.38E-76
FAM114A1	27.46	5.87	2.051	7.18E-90
RPE	20.4	4.15	2.055	7.22E-95
PSAP	844.702	176.998	2.248	2.84E-76
S100A10	1185.68	50.621	4.523	3.41E-120
FAM118B	15.13	2.73	2.112	6.94E-114
ERO1L	37.801	7.39	2.209	9.48E-76
S100A6	5221.685	53.891	6.572	3.84E-142
MPZL1	79.572	11.03	2.744	1.34E-106
SIRPA	22.68	4.65	2.067	1.64E-56
TOR4A	16.53	1.29	2.936	8.39E-100
SOWAHC	6.73	0.9	2.024	3.08E-106
TFDP1	35.439	7.63	2.078	3.61E-116
UBL5	407.285	85.781	2.234	3.88E-94
TXNRD1	31.349	5.47	2.322	5.56E-89
TPM2	246.133	31.349	2.933	9.10E-73
HLA-DRB5	62.391	1.19	4.855	5.58E-58
ITGBL1	39.289	1.37	4.087	1.09E-84
PPIAP22	196.678	17.74	3.399	6.87E-135
FAM3D	26.65	0.23	4.491	2.60E-73
GLMP	63	12.7	2.224	1.18E-103
PPP1R14C	5.85	0.19	2.525	1.75E-82
F5	16.26	0.6	3.431	3.45E-70
EPS8L3	56.621	0.31	5.459	1.66E-100
RCSD1	5.01	0.5	2.002	2.30E-58
TMEM184B	37.08	8.48	2.006	3.73E-87
PNP	64.431	7.43	2.956	6.70E-104
GRK5	12.36	1.66	2.328	8.95E-96
PRC1	17.259	3.52	2.014	1.62E-81
NAGA	25.34	3.38	2.588	9.20E-121
TGM2	151.271	8.62	3.984	6.46E-76
INF2	89.927	12.8	2.72	1.76E-81

SERTAD4-AS	4.97	0.29	2.21	9.74E-92
FCGR3A	37.869	1.85	3.77	4.90E-75
PPAPDC1A	9.34	0.04	3.314	1.93E-85
HIST2H2AA3	39.73	5.57	2.632	3.28E-37
RBM20	0.15	8.28	-3.012	3.87E-86
LIME1	20.26	3.07	2.385	8.46E-42
FAM127B	71.57	16.76	2.031	4.38E-89
MAFB	19.62	3.39	2.232	1.76E-50
HLA-DOA	12.46	0.69	2.994	5.29E-74
HNRNPCP2	10.09	0.51	2.877	4.30E-146
HLA-DMA	106.838	9	3.431	2.33E-85
PSMB8-AS1	21.98	2.58	2.682	3.57E-89
PSMB8	116.848	11.38	3.251	6.35E-105
TAP2	33.56	5.38	2.437	4.99E-84
LINC01420	38.799	5.01	2.727	2.38E-131
HLA-DRA	724.629	21.31	5.023	1.95E-100
LAYN	9.31	0.73	2.575	5.63E-91
SLC44A4	111.964	3.16	4.763	7.23E-102
HSPA1B	118.036	21.31	2.416	9.20E-20
HSPA1A	147.206	20.71	2.771	8.62E-26
LST1	36.041	1.48	3.901	1.98E-92
HLA-C	715.198	86.521	3.033	5.40E-88
PSORS1C1	8.14	0.39	2.717	7.27E-71
HLA-E	388.157	56.861	2.75	1.34E-88
TRIM15	8.02	0.01	3.159	2.83E-91
TRIM31	18.08	0.14	4.065	1.96E-83
HLA-F	148.158	11.77	3.546	2.72E-95
REG1P	0.22	140.789	-6.861	1.03E-58
MROH6	24.599	0.89	3.76	7.15E-99
IGFL2	14.65	0	3.968	1.40E-75
PRSS1	102.153	59372.201	-9.169	5.13E-59
PSENEN	123.647	25.24	2.248	1.71E-102
MUC12	4.63	0.09	2.369	2.45E-92
SARNP	67.751	15.6	2.05	7.24E-98
PRR13	241.502	49.869	2.253	5.36E-89
SAMD9	9.13	0.51	2.746	5.84E-103
KRT6A	9.47	0.7	2.623	2.88E-32
EXOC3L4	8.99	0.69	2.563	1.90E-58
TMSB4X	5503.37	636.403	3.11	6.42E-84
ITSN1	13.36	2.44	2.062	3.47E-81
SERPINB5	19.7	0.06	4.287	4.13E-100
HCP5	20.18	1.51	3.077	2.05E-93
HLA-H	52.301	2.67	3.86	1.11E-105
HLA-A	1174.555	61.311	4.238	5.41E-122
STK38L	19.87	3.99	2.064	2.58E-75
IGKC	2508.657	22.55	6.736	7.70E-76
IGKV4-1	59.26	0.57	5.262	4.00E-70

IGKV3D-20	3.69	0	2.23	3.68E-41
IGLV4-69	7.16	0.08	2.918	2.20E-49
IGLV8-61	6.73	0.16	2.736	4.77E-37
IGLV6-57	7.65	0.07	3.015	8.57E-55
IGLV1-51	28.829	0.88	3.988	3.17E-56
IGLV1-47	23.831	0.25	4.312	5.04E-61
IGLV7-46	8.02	0.17	2.947	4.63E-45
IGLV1-44	30.589	0.28	4.625	1.57E-59
IGLV7-43	5.44	0	2.687	3.71E-42
IGLV1-40	58.141	0.54	5.263	6.23E-65
IGLV3-25	24.38	0.18	4.427	3.16E-59
IGLV2-23	39.821	0.35	4.918	4.14E-72
IGLV3-21	13.9	0.13	3.721	2.63E-58
IGLV3-19	23.4	0.23	4.31	9.30E-60
IGLV2-14	75.441	1.35	5.024	3.65E-60
IGLV2-11	33.091	0.61	4.404	4.12E-60
IGLV3-10	7.24	0.14	2.854	2.53E-42
IGLV3-1	29.549	0.19	4.682	1.57E-67
IGLC1	323.113	3.55	6.155	3.41E-75
IGLC2	812.373	7.85	6.522	6.09E-73
IGLC3	361.762	3.9	6.21	7.06E-73
IGLC7	4.67	0	2.503	1.00E-40
TRBV25-1	14.41	0.51	3.351	3.93E-81
TRBC2	43.299	1.17	4.352	1.62E-91
IGHA2	160.986	3.6	5.138	5.97E-54
IGHG4	112.789	0.2	6.567	8.46E-73
IGHG2	167.741	1.17	6.281	2.51E-79
IGHA1	797.919	13.96	5.739	2.78E-62
IGHG1	886.329	3.9	7.501	2.11E-85
IGHG3	85.537	0.39	5.96	2.16E-77
IGHM	121.818	2.24	5.244	3.37E-65
IGHV6-1	3.76	0	2.251	2.63E-45
IGHV1-2	12.43	0.13	3.571	1.48E-46
IGHV1-3	3.53	0	2.18	2.61E-27
IGHV2-5	11.47	0.12	3.477	5.10E-54
IGHV3-7	18.9	0.27	3.97	3.62E-54
IGHV3-11	12.04	0.1	3.567	1.50E-53
IGHV3-15	17.97	0.23	3.947	7.32E-58
IGHV1-18	18.64	0.13	4.119	2.28E-59
IGHV3-21	23.41	0.16	4.395	7.86E-68
IGHV3-23	49.531	0.47	5.103	4.58E-59
IGHV1-24	6.56	0	2.918	3.94E-48
IGHV3-33	27.2	0.32	4.417	9.55E-59
IGHV4-34	9.1	0.11	3.186	5.88E-49
IGHV4-39	23.259	0.18	4.362	1.71E-60
IGHV1-46	4.34	0.06	2.333	2.93E-46
IGHV3-49	6.35	0.1	2.74	2.51E-47

IGHV5-51	25.69	0.18	4.499	2.18E-66
IGHV4-61	9.54	0.06	3.314	4.63E-54
IGHV1-69	3.78	0	2.257	3.88E-34
IGHV3-73	3.52	0	2.176	4.16E-40
RP11-641D5	290.037	59.141	2.275	2.05E-95
LINC00671	0.23	11.69	-3.367	2.98E-80
PTPRCAP	7.15	0.89	2.108	1.06E-49
HNRNPA1P33	5.8	0.06	2.681	8.61E-68
SIPA1	46.278	7.84	2.419	6.34E-84
VDAC1	177.896	34.75	2.323	9.50E-100
LBH	62.232	3.78	3.726	8.29E-104
PPP1CB	80.929	15.39	2.322	1.88E-87
SULT1A4	15.5	1.8	2.559	4.95E-77
GPSM3	31.18	2.14	3.357	1.95E-96
UBD	108.563	3.69	4.546	3.94E-62
ITGA1	14.26	1.55	2.581	1.80E-92
TAX1BP3	162.286	28.479	2.47	4.57E-86
UCA1	7.43	0.13	2.899	6.80E-53
SH3D21	5.24	0.44	2.115	4.87E-71
PLIN5	1.34	46.61	-4.347	1.24E-125
MEG3	29.65	149.758	-2.298	4.47E-46
MUC5AC	12.01	0.01	3.687	2.87E-50
GOLGA8B	12.33	155.588	-3.554	4.70E-103
TMED11P	0.14	72.028	-6.001	4.26E-92
MMP23A	6.92	0.66	2.254	2.64E-61
IFI30	235.583	20.069	3.489	3.53E-85
FAM19A5	9.14	0.91	2.408	1.44E-80
FTH1P8	10.26	1.13	2.402	1.81E-108
RP11-40C6	611.453	0	9.258	3.08E-186
TRIM16	26.6	3.02	2.779	6.86E-101
LINC00152	63.438	4.8	3.474	2.72E-103
RN7SKP54	7.71	0	3.123	7.79E-45
RP11-289I1	6.67	0.67	2.199	5.78E-98
HLA-DPB1	232.227	11.53	4.218	6.04E-97
UBE2SP2	5.69	0	2.742	2.61E-142
LINC01133	25.44	0.12	4.561	2.56E-89
IGHV4-59	16.959	0.21	3.892	6.11E-60
LINC01272	6.96	0.31	2.603	2.26E-75
IGHV3-74	9.35	0.2	3.109	1.77E-51
IGHV3-72	3.83	0.08	2.161	2.24E-40
TMEM191A	7.95	1.1	2.091	6.86E-86
FTH1P20	14.97	1.13	2.906	2.96E-119
MIR217HG	0.03	5.74	-2.71	1.31E-88
LINC00511	23.98	2.39	2.881	2.59E-105
RP11-395B7	6.06	0	2.82	6.45E-95
RPS28P7	1046.748	244.45	2.094	2.03E-76
LTB	17.3	0.63	3.489	3.75E-69

FLJ38122	0.12	8.98	-3.156	5.99E-114
RP1-241P17	6.93	0.65	2.265	2.07E-39
RP11-392O1	0.08	4.72	-2.405	7.94E-58
PGA4	0.1	4.99	-2.445	6.82E-27
HMGN1P37	3.89	0	2.29	2.54E-130
HLA-DRB6	19.149	0.3	3.954	6.71E-96
PGA3	0.14	6.84	-2.782	1.75E-35
TMSB4XP6	8.33	0	3.222	2.27E-06
RP4-631H13	0	4.05	-2.336	5.19E-26
HSBP1	90.283	17.23	2.324	1.77E-111
HLA-DPA1	192.898	8.45	4.359	4.48E-93
IGHV4-31	4.45	0	2.446	3.66E-43
TAPBP	96.348	17.31	2.411	1.05E-84
FTH1P7	26.521	1.74	3.328	5.88E-136
IGHV3-43	3.18	0	2.064	1.44E-41
RP11-14N7	10.16	1.17	2.363	5.92E-73
HLA-DQB2	8.11	0.14	2.998	3.39E-83
SLCO4A1-AS	3.83	0.17	2.045	3.27E-56
PHGR1	8.65	0.58	2.611	1.66E-37
HOXB-AS3	5.19	0.32	2.229	3.83E-82
GPX1	377.333	40.839	3.177	3.17E-103
LINC01237	0.37	5.77	-2.305	7.15E-96
LINC01503	8.98	0.48	2.753	1.71E-82
RP5-1108M1	0	11.78	-3.676	2.00E-10
RHOXF1P1	0.05	4.07	-2.272	7.10E-114
HLA-B	1432.579	99.85	3.829	2.95E-99
H3F3AP4	245.52	44.47	2.439	1.08E-73
HSPB1P1	6.12	0	2.832	5.45E-89
TRHDE-AS1	0.32	4.79	-2.133	3.53E-83
RP11-175B9	2.25	12.26	-2.029	3.59E-69
NR2F1-AS1	9.24	1.09	2.293	5.37E-77
UNC5B-AS1	3.5	0.11	2.019	3.25E-54
LINC00857	3.86	0.13	2.105	6.58E-95
HLA-DQA2	8.52	0.13	3.075	1.95E-58
KIFC1	6.12	0.22	2.545	6.22E-107
RP5-857K21	94.529	19.66	2.209	3.70E-39
OR2I1P	8.74	0.71	2.51	8.17E-37
IGKV1-6	7.4	0.12	2.907	6.65E-53
IGKV3-20	183.915	1.36	6.292	9.27E-74
PSMB9	81.718	4.03	4.04	3.02E-111
PCDHGC3	44.68	10.29	2.017	3.43E-67
RP11-331F4	1.47	3069.099	-10.28	1.04E-112
IGKV1-17	21.86	0.2	4.252	1.69E-59
RP11-680B3	0.05	31.95	-4.972	1.27E-107
IGKV1-8	11.88	0	3.687	1.32E-51
PNMA2	5.59	0.54	2.097	1.33E-65
TMEM189	33.4	5.23	2.465	6.43E-111

IGKV1-16	15.67	0.2	3.796	3.64E-57
IGKV2-24	10.71	0.26	3.216	3.93E-47
IGKV3-11	198.982	1.69	6.216	8.81E-78
RPL37P6	3.29	0	2.101	3.68E-100
PSMC1P1	20.99	3.26	2.368	7.66E-102
IGKV1-9	21.24	0.17	4.249	6.46E-61
PLEKHO2	16.52	2.25	2.43	2.41E-86
IGKV1-33	47.99	0.39	5.139	2.66E-65
UGT1A10	17.09	0	4.177	1.45E-78
IGKV2D-28	17.44	0	4.205	6.20E-52
HLA-DMB	63.03	3.42	3.857	3.23E-89
GNG10	48.271	6.84	2.652	2.12E-113
IGKV2-30	7.54	0.16	2.88	5.42E-43
IGKV2D-29	6.03	0	2.814	8.82E-40
PRAF2	25.849	3.67	2.523	1.45E-99
IGKV1-5	49.009	0.46	5.098	1.29E-69
TNFRSF6B	27.72	1.12	3.76	1.17E-58
RPLP0P2	3.86	0.1	2.143	1.22E-73
GSTA2	1.15	66.541	-4.973	8.49E-85
IGKV2-28	69.709	0.8	5.296	2.50E-63
IGKV3-15	54.651	0.44	5.272	6.57E-69
RP11-1398P	4.76	0.31	2.137	8.54E-99
SCARF2	12.2	1.78	2.247	2.43E-63
RP11-10G12	4.62	0	2.491	1.58E-124
IGKV1-27	16.54	0.22	3.846	6.48E-51
MIR210HG	12.79	1.74	2.331	1.12E-47
TWF2	56.861	6.26	2.995	6.34E-113
MTND4P12	17.1	0.18	3.939	9.09E-36
PCED1B-AS1	13.41	1.15	2.745	9.70E-69
ECSCR	13.9	2.18	2.228	3.20E-74
PVT1	12.5	0.62	3.059	5.05E-64
RP11-256P1	3.4	0	2.138	3.33E-36
TMEM158	8.02	0.6	2.495	9.93E-78
IQCJ-SCHIP	8.1	0.52	2.582	7.47E-96
PRSS3P1	0.04	15.6	-3.997	1.13E-68
RP11-834C1	11.99	1.41	2.43	1.61E-69
IGKV1D-39	102.089	0.88	5.777	2.63E-69
RNA5SP216	0	3.79	-2.26	1.72E-15
TRNP1	39.681	7.4	2.276	3.23E-63
HOXA-AS2	7.35	0.74	2.263	4.66E-75
IGHGP	16.45	0	4.125	2.02E-72
LYN	19.49	3.37	2.229	9.06E-77
RP11-719K4	17.23	0.78	3.356	1.40E-58
RP11-468E2	12.57	0.29	3.395	1.19E-81
IGLL5	34.071	0.37	4.678	7.03E-67
RP11-805J1	11.44	1.06	2.594	1.89E-123
SLC22A18AS	3.94	0.07	2.207	1.61E-90

RP11-326C3	11.19	257.315	-4.405	9.59E-70
PIGY	13.18	0.2	3.563	6.49E-52
RP11-350N1	0.42	5.95	-2.291	5.71E-101
RP4-809F18	0.17	4.35	-2.193	9.14E-88
TRAPPC2B	14.69	2.5	2.164	2.75E-115
RP11-320N7	0.26	15.07	-3.673	1.54E-66
GALNT4	13.81	1.2	2.751	2.86E-77
RP11-1100I	17.72	3.47	2.066	1.65E-79
RP11-1143(	52.359	0.48	5.172	2.14E-50
RP11-386G1	73.312	6.01	3.406	4.18E-33
RP11-161H2	123.613	9.85	3.522	1.21E-100
RP11-649E7	4.54	0.18	2.231	5.89E-38
RP11-638I2	13.04	1.13	2.721	2.11E-74
TUBB3	35.7	0.67	4.458	3.04E-112
RP11-316M1	11.55	0.19	3.399	1.36E-61
SORD2P	8.06	1.24	2.016	8.89E-74
SLC22A31	0.25	4.73	-2.197	1.70E-37
RP11-1100I	6.33	0.61	2.187	2.79E-29
RP11-304L1	6.16	0.27	2.495	8.37E-76
HOXB7	16.59	0.42	3.631	2.19E-109
MRC1	8.56	0.98	2.272	3.56E-50
RP11-211G2	3.09	0	2.032	1.88E-50
WFDC21P	7.57	0.38	2.635	5.18E-71
RP11-304L1	5.25	0.45	2.108	7.67E-67
PECAM1	36.161	6.15	2.378	6.12E-69
RP11-345J4	27.54	4.74	2.314	3.37E-109
RP11-626G1	25.46	5.09	2.119	9.08E-77
MIA	54.78	0.41	5.306	2.00E-88
MMP12	5.57	0	2.716	1.50E-64
RP11-667K1	5.65	0	2.733	3.02E-75
SPON1	18.23	1.8	2.78	7.56E-64
RP11-498C9	3.09	0	2.032	1.06E-26
RP11-334E6	57.651	1.37	4.629	2.00E-102
RP11-746M1	18.03	3.44	2.1	9.09E-27
RP11-6N17	4.59	0.34	2.061	9.62E-45
SNRPGP2	10.69	1.12	2.463	8.39E-95
RBM8A	121.153	23.3	2.33	9.86E-103
TIMM23	46.85	10.88	2.01	3.12E-113
RP11-138I1	126.956	23.88	2.363	5.03E-88
SRGAP2	16.04	3.2	2.02	5.65E-74
PNLIPRP2	4.11	2034.698	-8.638	2.15E-67
GDF10	0.23	4.57	-2.179	5.76E-102
RP11-78A19	15.82	1.1	3.002	9.70E-94
LUZP6	18.69	2.07	2.681	2.34E-33
RP11-64C12	0.1	7.66	-2.977	4.32E-131
SMIM22	274.658	57.619	2.233	7.19E-60
SLC6A14	11.3	0.01	3.606	1.23E-77

RP3-461F17	12.16	2.14	2.067	7.15E-105
RP5-940J5	6.03	0	2.814	1.23E-31
IGHV3-30	41.729	0.41	4.921	1.52E-62
MMP28	36.269	0.58	4.56	1.43E-118
LIX1L	13.03	2.07	2.192	5.63E-101
MILR1	6.12	0.64	2.118	6.00E-66
HIST2H2AA4	21.35	3.12	2.44	5.57E-27
RP11-284F2	3.43	0.07	2.05	8.56E-67
RP4-583P15	16.26	0.25	3.787	2.10E-106
RP11-350J2	18.191	0	4.262	1.10E-94
RN7SL386P	0	72.308	-6.196	1.12E-128
TP53TG1_1	4.52	0.29	2.097	5.37E-10
IGHV2-70	3.31	0	2.108	5.30E-36
SGK223	8.94	1.06	2.271	1.50E-84
PRSS2	449.319	83831.219	-7.54	1.84E-56
UHRF1	4.56	0.12	2.312	1.08E-103
ORAI1	18.21	3.64	2.05	2.82E-99
TP53TG1_2	6.34	0.13	2.699	3.40E-22
RAB7B	6.07	0.33	2.41	2.59E-88
IGHV4-4	4.73	0	2.518	1.42E-32
TRAC	23.33	0.82	3.741	1.01E-83
PSMB3	190.229	31.299	2.566	3.48E-111
RP11-452N1	44.359	4.92	2.938	4.14E-97
RN7SL186P	0.11	153.234	-7.118	3.82E-133
IGLV2-8	20.33	0.45	3.879	4.25E-54
RP4-738P15	11.86	0.72	2.902	2.23E-36
RP11-671J1	29.829	4.72	2.43	2.01E-110
IGHV1-69-2	21.63	0.13	4.324	1.85E-62
U91328	3.78	0	2.257	6.13E-21
TGFB2-OT1	5.4	0.59	2.009	1.99E-49
TMEM265	42.461	3.48	3.278	1.83E-94
TMBIM4	0	5.14	-2.618	6.12E-14
